# The choice of the DNA extraction method may influence the outcome of the soil microbial community structure analysis

**DOI:** 10.1002/mbo3.453

**Published:** 2017-02-20

**Authors:** Sylwia Zielińska, Piotr Radkowski, Aleksandra Blendowska, Agnieszka Ludwig‐Gałęzowska, Joanna M. Łoś, Marcin Łoś

**Affiliations:** ^1^ Department of Molecular Biology University of Gdansk Gdansk Poland; ^2^ Center for Medical Genomics – OMICRON Faculty of Medicine, Jagiellonian University Medical College Kraków Poland

**Keywords:** 16S rDNA, commercial kits, DNA extraction, microbial community, NGS library, soil sample

## Abstract

Metagenomics approaches and recent improvements in the next‐generation sequencing methods, have become a method of choice in establishing a microbial population structure. Many commercial soil DNA extraction kits are available and due to their efficiency they are replacing traditional extraction protocols. However, differences in the physicochemical properties of soil samples require optimization of DNA extraction techniques for each sample separately. The aim of this study was to compare the efficiency, quality, and diversity of genetic material extracted with the use of commonly used kits. The comparative analysis of microbial community composition, displayed differences in microbial community structure depending on which kit was used. Statistical analysis indicated significant differences in recovery of the genetic material for 24 out of 32 analyzed phyla, and the most pronounced differences were seen for *Actinobacteria*. Also, diversity indexes and reproducibility of DNA extraction with the use of a given kit, varied among the tested methods. As the extraction protocol may influence the apparent structure of a microbial population, at the beginning of each project many extraction kits should be tested in order to choose one that would yield the most representative results and present the closest view to the actual structure of microbial population.

## 1 Introduction

Due to the lack of ability to culture almost 99% of bacteria using traditional microbiology methods, extraction of bacterial DNA directly from environmental samples has become a method of choice in the environmental microbiology studies. This kind of approach allows obtaining previously unknown bacterial DNA and thus it can lead to discovery of novel genes, e.g., encoding proteins with a desired function, such as resistance to antibiotics or involved in pollutant degradation (Handelsman et al., 2007). This is also a useful tool when it comes to estimating a population diversity in a particular environment (Daniel, 2005; Zhou, Bruns, & Tiedje, 1996), as vast majority of bacteria may be omitted when using traditional cultivation methods.

The DNA extraction protocol can be crucial when attempting to isolate the most representative environmental DNA sample. It has been shown that due to differences in the bacterial cell wall and membrane structures, the effectiveness of DNA extraction can depend on the procedure used (Carrigg, Rice, Kavanagh, Collins, & O'Flaherty, 2007; Felczykowska, Krajewska, Zileińska, & Łoś, 2015; Krsek & Wellington, 1999), while successful application of molecular techniques relies on an efficient recovery of nucleic acids from environmental samples (Hurt, Qiu, Wu, Roh, & Palumbo, 2001). Thus, it is important to choose methods that yield both, good quality and high quantity of the extracted DNA.

Soil is often considered as one of the most challenging environments, mostly due to the diversity of the species present and the variety of enzymatic inhibitors (like humic acids or heavy metals) which can be co‐extracted with DNA. It can also contain mineral particles of different size or origin, and organic compounds at various stages of decomposition (Daniel, 2005). In the past, many reports focused on comparing the soil DNA extraction methods, both direct and indirect ones (Gabor, de Vries, & Janssen, 2003; Islam, Sultana, Joe, Cho, & Sa, 2012; Krsek & Wellington, 1999; Robe, Nalin, Capellano, Vogel, & Simonet, 2003). Currently, many commercial DNA extraction kits are available and due to their efficiency and short time of extraction they are replacing the traditional extraction protocols. Often, more consistent results with repeated sampling are obtained when using the extraction kits instead of traditional extraction protocols. This aspect is important, especially when the goal of a study is to track differences across environments, treatments, or timescales (Morgan, Darling, & Eisen, 2010).

Most of the commercial DNA extraction kits are based on direct extraction methods and their components are the trade secret. Different procedures and buffers used for the DNA extraction and purification can cause differences in quantity and purity of the genetic material obtained. Often, many studies focus on comparing the recovered amount of DNA and its purity from different types of soil. However, an important issue is how the amount and good quality of the extracted DNA relates to the diversity of the extracted microbial DNA and in consequence how the diversity of the sequences obtained reflects a given species' presence, as large amount of DNA is not equivalent to its diversity.

Until now, several studies has been conducted in order to investigate presented issue with the use of different extraction methods, as well manual protocols and commercial kits. In order to compare microbial community structure, investigated with the use of different extraction methods, authors used different microbial community analysis methods. Stach, Bathe, Clapp, and Burns (2001) performed a PCR‐SSCP analysis (PCR‐single strand conformation polymorphism) for a silty loam soil sample. That study compared different direct methods of DNA extraction and purification, and also investigated the relationship between the DNA quantity and the sequence diversity. That analysis had demonstrated distinct differences in sequence representation between the extraction methods used and had indicated that a higher DNA yield is not synonymous with higher sequence diversity. This implies that the DNA extraction methods should be evaluated not only in terms of the quantity and purity of the material to be obtained but also in terms of a given method's influence on the sequence diversity. Martin‐Laurent et al. (2001) used amplified ribosomal DNA restriction analysis (ARDRA) and ribosomal intergenic spacer analysis (RISA) in order to demonstrate that soil DNA extraction methods can affect both phylotype abundance and composition of the indigenous bacterial community. Feinstein, Sul, and Blackwood (2009) with the use of qPCR, analysis of T‐RFLP profiles (terminal restriction fragment length polymorphism) and pyrosequencing suggested that DNA extraction bias can be greatly reduced for some analyses by pooling three successive extractions as the majority of DNA is obtained within the first few extractions. Delmont et al. (2011) used i.a. pulsed‐field gel electrophoresis and RISA analysis and suggested that using different metagenomic approaches will maximize the representation of different species in microbial community, but can distort perception of relative microbial abundance. However, the real microbial community structure remains unknown and most methods provide only limited views of the true soil biodiversity, thus using multiple metagenomic methods offer more complete view. Additionally, Morgan et al. (2010) by creating and testing in vitro‐simulated microbial community suggested using multiple DNA extraction procedures with a single environmental sample in order to increase the likelihood of discovering every organism in the tested sample. Authors, with the simple test, demonstrated that two libraries created from a single mixture of organisms, prepared with DNA extracted by different protocols, can produce reads, suggesting to represent two various communities. Therefore, it has to be established at an early stage of each study as to which extraction protocols to choose in order to obtain DNA from the target group of organisms.

Selection of the optimal method is of a high importance and it can influence the results and its interpretation. This is why the aim of this paper is to compare the efficiency, quality, and most of all diversity of genetic material extracted with the use of commonly used commercial DNA extraction kits. We want to investigate whether the DNA extraction methods affect the outcome of the microbial communities' genetic structure analyses and diversity of the sequences obtained. As the soil is very often described as the most challenging of all natural environments (Daniel, 2005), we decided to perform our comparative analysis on one selected soil sample in order to establish if 16S rDNA Next Generation Sequencing (NGS) libraries created based on DNA isolated with the use of different extraction kits will produce reads suggesting to represent the same or various microbial communities. Until now, several papers focused on such comparisons but with the use of different approaches. In here, we present new insights into the old issue.

## 2 Experimental Procedures

### 2.1 Sample collection

Soil samples were collected in the northern part of Poland (Wiślinka; 54° 19′ N, 18° 50′ E) at the beginning of September 2015, in an area adjacent to a phosphogypsum waste heap. Since 1969, the selected area in Wislinka, Pomerania district, became a landfill of post‐production wastes. The size of the landfill including its security zone amounts to 85 ha, of which 26 ha are taken up by the waste heap (Hupka et al., 2006). Currently, this area is subjected to reclamation by a cover of vegetation and sludge from wastewater treatment plants, which are characterized by the presence of heavy metals, such as copper, zinc, cadmium, lead, chromium, and nickel. A number of expertise reviews were conducted in order to determine the impact of this landfill on the environment (Boryło & Skwarzec, 2014; Skwarzec & Boryło, 2013; Skwarzec, Boryło, Kosińska, & Radzajewska, 2010).

The soil sample was collected after ground vegetation removal from an area covering one square meter, with the use of Eijkelkamp soil collection rings; eight ring samples combined and randomly put together, mixed and sieved to form a composite sample. Plant roots were removed from the soil. The depth of the soil layer was ranging from 0 to 10 cm. The soil sample was immediately transported (within 1 hr of collection) in a sterile bowl for sieved soil, to a lab facility where it was divided into small portions for the DNA extraction by protocols according to the manufactures' instructions.

### 2.2 DNA extraction

DNA was extracted from the soil sample with the use of eight commercial kits, according to the manufacturers' protocols (Kit's company number – shortcut use in the paper). Details about the kit names and companies are available upon request.

1. Company 1 – C1
2. Company 2 –C2
3. Company 3 – C3
4. Company 4 – C4
5. Company 5 – C5
6. Company 6 lysis buffer 1 –C6.1
7. Company 6 buffer 2 – C6.2
8. Company 7 –C7
9. Company 8 – C8

The DNA extraction kit from company 6 has two different lysis buffers that can be used for the DNA extraction, and as recommended by the manufacturer's instructions, both lysis buffers should be tested in parallel with every new soil sample. When required by the protocol, FastPrep^®^ Instrument (MP Biomedicals, Santa Ana, CA) was used.

To avoid cross contamination of the samples, the process was performed with sterile equipment. The quantity and quality of the extracted DNA were evaluated by using a Nano Drop spectrophotometer. We also evaluated DNA extraction kits according to the convenience of their use, time spent on extraction, and cost per sample. In each category, we ranked the kits on a scale of 1–8, where 1 means the best in the category and 8 means the last in the category. In case of kit C6 that includes two different lysis buffers, we evaluate extraction procedure only once, due to the same lab procedure, at a later stages we consider them as separate kits (C6.1 – lysis buffer 1, C6.2 ‐ lysis buffer 2). We also present a final ranking of the kits in all categories. It is worth to mention that these estimates represent a subjective opinion of the user. After extraction, the DNA was stored at −20°C for further use.

### 2.3 16S rDNA amplification and sequencing

The V3‐V4 hypervariable regions of bacterial 16S rDNA were amplified using the following primer set: 341F ‐ CCTACGGGNGGCWGCAG and 785R ‐ GACTACHVGGGTATCTAATCC. The targeted gene region has been shown to be the most appropriate for the Illumina sequencing (Klindworth et al., 2013). Each library was prepared in a two‐step PCR protocol, based on Illumina's “16S metagenomic library prep guide” (15044223 Rev. B) using the Q5 Hotstart High‐Fidelity DNA Polymerase (NEBNext ‐ New England BioLabs) and a Nextera Index kit. Paired‐end (PE, 2 × 250nt) sequencing with a 5% PhiX spike‐in was performed with an Illumina MiSeq (MiSeq Reagent kit v2) at Genomed, Warsaw, Poland, following manufacturer's run protocols (Illumina, Inc., San Diego, CA, USA). The automatic primary analysis and the de‐multiplexing of the raw reads were performed on MiSeq with the use of MiSeq Reporter (MSR) v2.4 software (BaseSpace).

### 2.4 Sequencing data analysis and statistical analysis

Samples were processed and analyzed using the Quantitative Insights Into Microbial Ecology (Qiime) pipeline v 1.9.1 software (Caporaso, Bittinger, et al., 2010; Caporaso, Kuczynski, et al., 2010) . Low‐quality PE reads (Andrews, 2010) and adapter sequences (Martin, 2011) were removed before further analysis. Quality‐filtered reads were merged based on the overlap of PE read with the use of fastq‐joint (Aronesty, 2011). The remaining sequences that did not meet the quality criteria were removed from further analysis (mean quality >20). Clustering of operational taxonomic units (OTUs) at 97% similarity was performed by using the uclust method version 1.2.22q (Edgar, 2010). OTUs were assigned to taxa using the GreenGenes release 13.08 (DeSantis et al., 2006) as a reference, with a taxonomy assignment tool PyNAST (Caporaso, Bittinger, et al., 2010; Caporaso, Kuczynski, et al., 2010). The Biological Observation Matrix (BIOM) table was used as the core data for downstream analyses (McDonald et al., 2012) and vsearch 1.7.0 (VSEARCH GitHub website: https://github.com/torognes/vsearch) as OSS replacement of usearch 6.1. Based on clusters, the diversity indices were estimated, including the Chao1, PD (a quantitative measure of phylogenetic diversity), Shannon, and Simpson indices, and also observed OTUs. Comparison of the microbial community structures was performed with the use of UniFrac (Lozupone & Knight, 2005) and Emperor (Vázquez‐Baeza, Pirrung, Gonzalez, & Knight, 2013). For OTU frequency comparison, the Kruskal–Wallis one‐way analysis of variance was performed, with p‐value estimated using the Fisher Z transformation based on metadata associations – MaAsLin Tickle, T., Waldron, L., Yiren, L., Huttenhower, C, in prep. The NGS data are deposited and fully available under study accession number PRJEB12454 in ENA – the European Nucleotide Archive.

## 3 Results

### 3.1 Evaluation of DNA extraction kits

For all tested DNA extraction kits, the amount and quality of the obtained DNA was established and is presented in Table 1. We were able to extract the highest amount of good quality DNA with Kit C7, but standard deviations counted from extraction repeats were also very high. On the other hand, we were able to extract a large amount of good quality DNA with reproducible results when using Kit C5. None of the DNA samples had brownish color, characteristic of the presence of humic acids. In all extraction methods tested, there were no PCR inhibitors in the DNA sample, as in all cases we obtained a DNA amplification product.

**Table 1:** The quantity and quality of the extracted DNA

Kit ID	μg of DNA per 1 g of soil	260/280	260/230
C1	1.01 ± 0.72	1.24 ± 0.97	0.48 ± 0.12
C2	1.84 ± 0.52	1.81 ± 0.39	0.07 ± 0.05
C3	3.52 ± 3.26	1.62 ± 0.24	0.29 ± 0.17
C4	0.63 ± 0.42	1.41 ± 0.34	0.43 ± 0.07
C5	3.20 ± 0.77	2.30 ± 0.46	0.03 ± 0.01
C6	1.99 ± 0.89	1.69 ± 0.18	0.47 ± 0.20
C7	6.00 ± 6.09	1.42 ± 0.24	0.63 ± 0.11
C8	0.89 ± 0.39	1.81 ± 1.09	0.33 ± 0.16

Results shown are mean values and standard deviation calculated for 10 replicates of each isolation. The color of all tested samples was clear. PCR products were obtained for DNA samples obtained with all tested kits.

Among the eight tested commercial kits, in our evaluation considering the convenience of use, time spent on extraction and cost per sample, two kits worked the best: C2 and C3, while C7 was ranked at a third place. Detailed classification and the results for each category are available in Table S1.

### 3.2 General description of sequencing results

**Figure 1:**
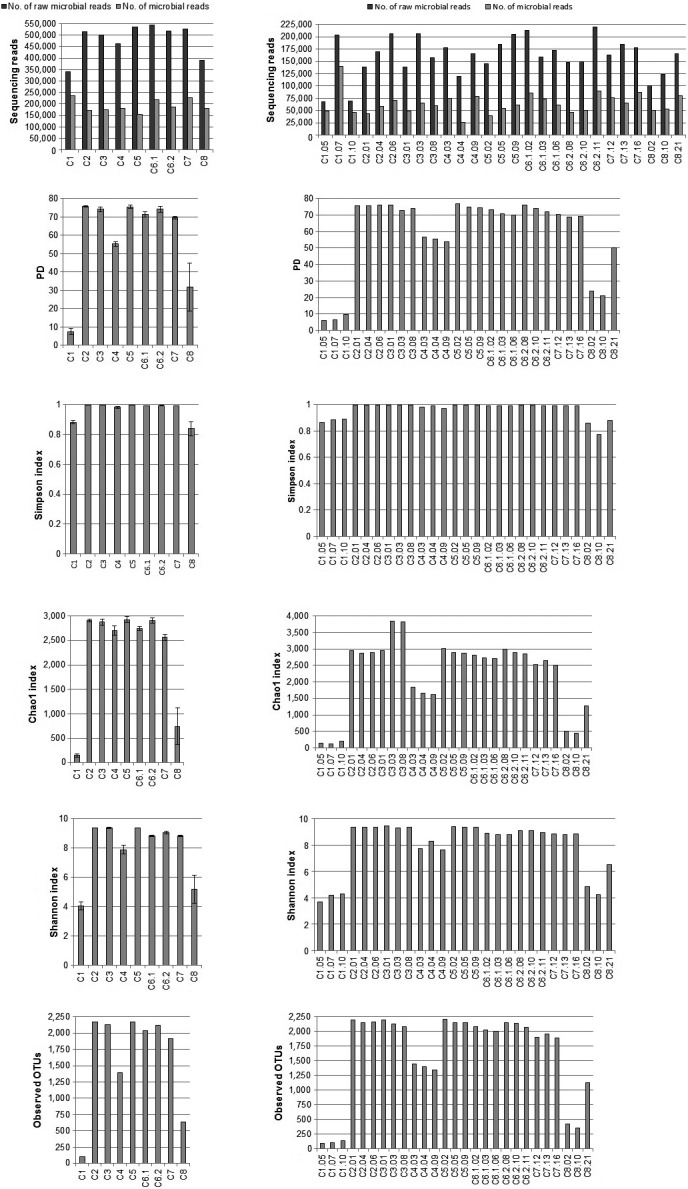
Summary of the sequencing data and statistical analysis of microbial community structures. (a) Summary of the extraction methods used, (b) data for three replicates of each extraction kit used. The ID abbreviations are defined in the text. The number of OTUs (operational taxonomic units) was generated at the 97% sequence similarity cut‐off. Diversity indices represent the randomly selected subsets for each sample normalized to 26090 sequences

**Figure 2:**
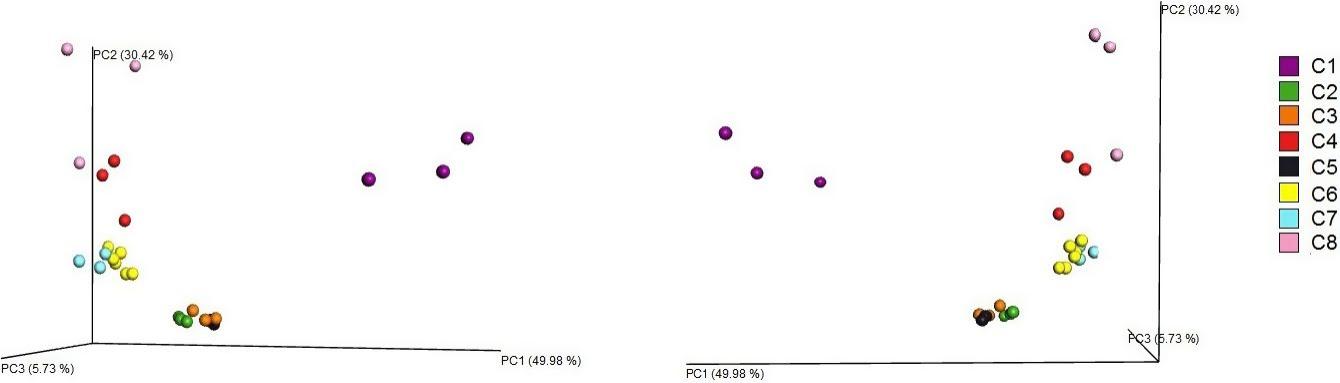
Comparison of the microbial community structures with the use of the principal coordinate analyses of Bray–Curtis dissimilarities (weighted unifrac) at the phylum level in all replicates of the tested extraction kits

With the use of different extraction kits, on average we obtained 173 039 good quality 16S rRNA gene sequences (V3‐V4 region), ranging between 155 099 (C5) and 235 590 (C1). When counting single replicates of different extraction kits we obtained range, 26 099 (C4) – 140 354 (C1) of good quality sequencing reads (V3‐V4 region). More details for sequence data for both, the kit analysis and single replicates among kits, are shown in Figure 1 (part a and b, respectively). At the phylum level, we were able to classify all of the sequences obtained. Figure 1 presents the number of the observed OTUs and the diversity indices for each extraction kit (a) and for replicates among kits (b). Principal coordinate analysis performed to compare the apparent compositions of microbial communities among kits is presented in Figure 2. Detailed taxonomic analyses on different ranks are available in supplementary data as sunburst charts for mean values of each kit (Figure S1) and also in a table (Table S2).

### 3.3 Microbial community composition

The analysis of microbial communities indicated that for all of the tested extraction kits, generally more than 99.97% of the total reads were represented by *Bacteria* (Figure S1 and Table S1). The highest percentage of *Bacteria* was assessed with the use of the C1 kit and the smallest with the C2 kit. Among replicates, the highest percentage of *Bacteria* was also assessed with the C1 kit and the smallest with the C2 kit. Taxonomy‐based analysis of the soil sample with the use of different extraction kits generally indicated that the microbial community being investigated consists of 33 phyla and 13 of them were common for all extraction kits, while 20 of them are absent in the DNA sample obtained with the C1 kit. For the rest of the tested kits, 3 to 7 phyla are absent, mostly those of low percentage of participation in the total share of the microbial community.

**Figure 3:**
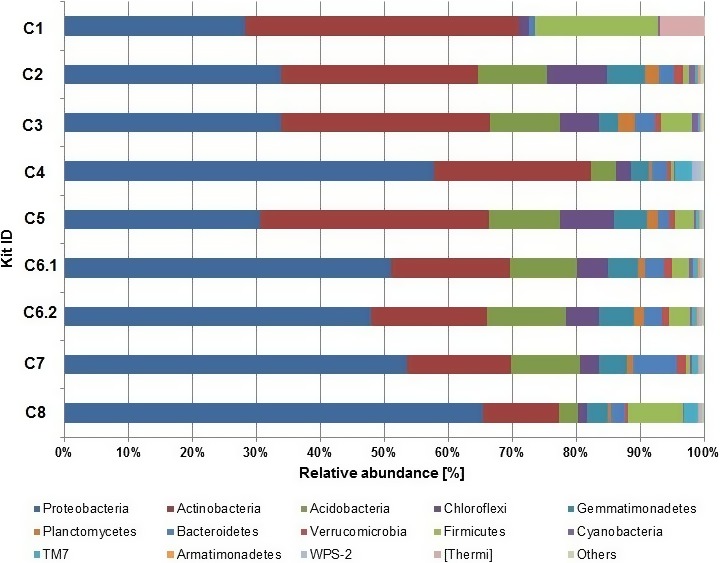
Abundance of microbial 16S rDNA sequences at the phylum level. Analyses of the microbial community structures for the analyzed extraction kits. “Other” describes: TM6, Nitrospirae, OD1, Chlorobi, Crenarchaeota, Elusimicrobia, Planctomycetes, Fibrobacteres, FBP, MVP‐21, Tenericutes, WS2, AD3, Chlamydiae, BRC1, OP11, Spirochaetes, Fusobacteria, Euryarchaeota, FCPU426

**Figure 4:**
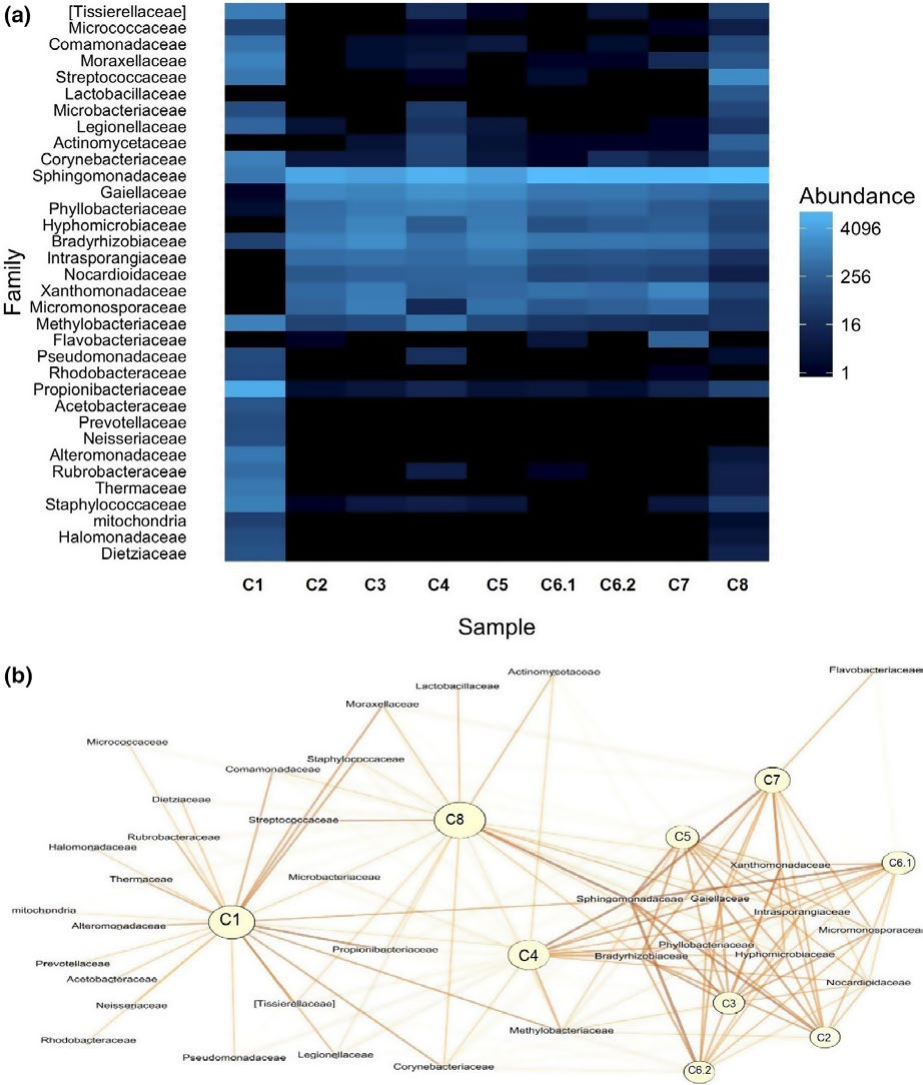
Comparing similarities in microbial population structures created with the use of different extraction kits. (a) The cluster heatmap display of relative abundances at the Family level of the microorganisms in DNA samples obtained with a given kit. Higher abundance is shown as lighter color. (b) Gephi scheme presents clustering of the used extraction kits. Relative abundances of microorganisms at the Family level in DNA samples obtained with a given kit. Higher abundance is shown as intense color. Higher aggregation of kits suggests more similar microbial structure

The most abundant phyla across all tested DNA extraction kits were *Proteobacteria*,* Acidobacteria*, and *Actinobacteria* (Figure 3, Table S2, Figure S2). Those phyla jointly accounted for more than 71.08% (C1) to 86.21% (C4) of the total microbial sequences obtained. Separately, *Proteobacteria* comprised on average 44.65%, in the range from 28.15% (C1) to 65.44% (C8), *Actinobacteria* comprised on average 25.76%, in the range from 11.89% (C8) to 42,93% (C1), and *Acidobacteria* comprised on average 8.13%, in the range from 0.01% (C1) to 12.29% (C6.2) of the total reads (Figure 3). The remaining reads in the population structure were associated with: *Chloroflexi*,* Gemmatimonadetes*,* Planctomycetes*,* Bacterioidetes*,* Verrucomicrobia*,* Firmicutes*,* Cyanobacteria*, TM7, *Armatinonadetes*, WC7‐2, TM6, *Nitrospirae*, OD1, *Chlorobi*,* Crenarchaeota*,* Elusimicrobia*,* Fibrobacteres*, FBP, MVP‐21, *Tenericutes*, WS2, [Thermi], AD3, *Chlamydiae*, BRC1, OP11, *Spirochaetes*,* Fusubacteria*,* Euryarchaeota*, and FCPU426, with different contribution to the population (Figure 3, Figure S1, Table S2). For each of the tested extraction kit, at least 10 phyla (up to 12) were responsible for more than 99.0% of the total microbial population. Similarities between the microbial community structures, taking into account the different extraction protocols, are illustrated with a heatmap and a Gephi scheme demonstrating the abundance of microorganisms at the family level for each kit (Figure 4, part a and b, respectively).

The Kruskla–Wallis test and *p*‐value, at the phylum level, indicated a significant difference between extraction kits for 24 out of 32 analyzed phyla (Table S3). No significant differences were found for *Fibrobacteres*,* Cyanobacteria*, AD3, *Fusobacteria*, [Thermi], *Euryarchaeota*, BRC1, *Spirochaetes*. The most visible differences between the extraction protocols were seen for *Actinobacteria*.

Microbial population structures with the most different composition, in comparison to all extraction kits tested, were those extracted with the C1 and C8 kits, especially when comparing the phyla present at a lower abundance level (Table S2, Figure 3, Figure S1). Average share of the [Thermi] phylum is 0.79% for all the kits tested, but for the C1 kit the percentage of its microbial contribution is 6.97%. In addition, the *Thermus* genus*,* for this extraction kit, was the only detected genus. In other kits, contribution of the [Thermi] phylum is often a mix of two genera: *Deinococcus* and *Thermus*.

Using different extraction kits, various contributions can be observed at lower taxonomic levels. Analyzing the *Proteobacteria* phylum, we can observe different contribution of the *Alphaproteobacteria* class with an average share of 33.46%, in the range from 10.34% (C1) to 59.10% (C8). For *Gammaproteobacteria*, average share is 6.70% with the range from 3.51% (C2) to 13.73% (C1). For *Betaproteobacteria* and *Deltaproteobacteria,* the differences are not so divergent, i.e., they were on average 3.80%, in the range from 1.74% (C8) to 5.75% (C7), and on average 1.19% in the range of less than 0.01% (C1) to 2.35% (C7), respectively. For *Acidobacteria*, the *Acidobacteriia* class for the seven tested kits constitutes around 5% of the total population, but with the use of the C1 kit it seems to constitute less than 0.01%, and 0.77% with the use of C4 and 1.02% with the use of the C7 kit. Similarly, for the *Solibacteres* class, in the case of seven tested kits, its contribution is around 3% and for the C1 kit it is less than 0.01%, for C4 it is 0.53%, and for C8 it is equivalent to 0.57% of the total population. When analyzing *Actinobacteria*, the *Actinobacteria* class constitutes on average 16.04%, in the range from 7.59% (C8) to 40.12% (C1). The *Rubrobacteria* class, for eight analyzed extraction kits, shares less than 0.07% of the total number of reads, while for the C1 kit it shares 2.80%. It can be also observed that in this particular soil sample, both lysis buffers tested with kit C6 gave comparable results.

**Figure 5:**
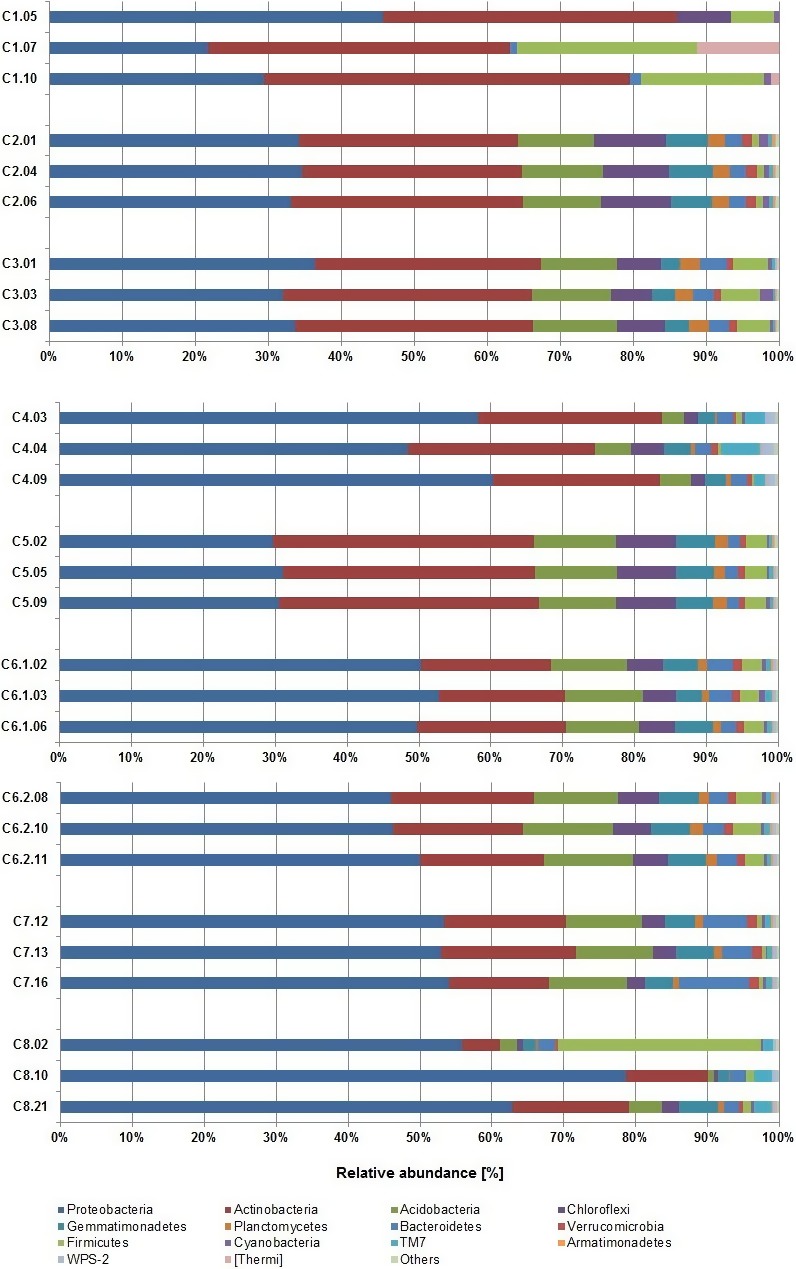
Abundance of microbial 16S rDNA sequences at the phylum level. Analyses of the microbial community structures for three replicates among analyzed extraction kits. “Other” describes: TM6, *Nitrospirae*, OD1, *Chlorobi*,* Crenarchaeota*,* Elusimicrobia*,* Planctomycetes*,* Fibrobacteres*, FBP, MVP‐21, *Tenericutes*, WS2, AD3, *Chlamydiae*, BRC1, OP11, *Spirochaetes*,* Fusobacteria*,* Euryarchaeota*, FCPU426

Among replicates of the extraction kits, different levels of reproducibility can be observed (Figure 5, Figure S1, Table S2). Kits C2, C3, C6.1, C6.2, and C5 are characterized by high reproducibility of results between replicates, even for the phyla with a lower contribution to the microbial community structure. The *Chloriflexi* phylum, in two replicates of kit C1, share less than 0.01% of the total microbial population and in one replicate the share is higher than 7%. With the same kit, when looking at *Firmicutes*, the share of this phylum in subsequent replicates is 5.91%, 16.87%, and 24.68%. Also, the already mentioned [Thermi] in one replicate constitute 0%, in the second 1.18%, and in the third even 11.31% of the total population, resulting in high contribution of that phylum when considering average values obtained for this kit. For kit C8, *Firmicutes* also constitute less than 1.3% of the total population in two replicates, but in the third replicate their contribution to the whole population is almost 30%, also resulting in a high contribution of that phyla, when considering average values obtained with this kit. For kit C4 we can observe differences in abundance of the TM7 candidate division; in each replicate they contribute accordingly: 1.46%, 5.37%, and 2.71%.

## 4 Discussion

Due to influence on the results obtained and their interpretation, selection of appropriate methods is of high importance in every research. Differences in cell wall and cell membrane structures of microorganisms can affect the effectiveness of a given DNA extraction protocol (Carrigg et al., 2007; Krsek & Wellington, 1999). Moreover, it has been shown, especially for the problematic soil samples, that when using different extraction procedures, varied amounts and quality of the DNA may be obtained (Gabor et al., 2003; Islam et al., 2012; Krsek & Wellington, 1999; Robe et al., 2003), as different soil microorganisms have different susceptibilities to various cell lysis methods (Daniel, 2005). On the other hand, even if the DNA is recovered from the soil sample, it may be useless for further reactions, due to humic acids or other enzymatic inhibitors that can be co‐extracted in the DNA sample. In this study, we extracted varied amounts of DNA from a single soil sample with the use of eight different commercial kits (additionally, for C6 we used two lysis buffers C6.1 and C6.2; Table 1). All DNA samples exhibited a different level of purity, but in every case the PCR reaction could be performed and there was no brownish color, characteristic of the presence of humic acids.

However, in practice, other aspects than quality and amount of recovered material are equally important when choosing an extraction protocol, like convenience of use and the time needed for the DNA extraction, as well as a cost per sample, which can be easily compared between each other. These parameters are very important criteria in extraction protocol selection and can be very subjective, but there are more important parameters, like sequence diversity and sequence representation. This can have a significant impact on the proper assessment of the final structure of microbial communities. With the use of PCR‐SSCP analysis, differences in sequence representation were already shown between the extraction methods (Stach et al., 2001). It was also demonstrated that the DNA extracted from a single mixture of organisms with the use of different protocols can produce reads that seem to represent different community structures (Morgan et al., 2010). In this work, we present the analyses of microbial community structure with application of 16S rDNA and the use of NGS that give a complex picture as to how microbial community structure can depend on the DNA extraction protocol.

In this study, we observed a high number of good quality reads above 150,000 in the kit analysis, and above 26,000 for single replicates among the tested kits. Generally, 15,000–100,000 reads per sample are sufficient for classification, as described in the Illumina 16S Metagenomic Sequencing Protocol. Also, in each analysis we were able to classify all of the obtained sequences at the phylum level. For each extraction kit, we observed different values for the diversity indexes. The high and similar values of the Shannon's and Simpson's indexes, not only in the case of kit C2 and C5, but also C3 and C6.2, suggest a high level of the species diversity estimated with the tested kits, and also indicate a similar diversity in the obtained populations. Values for each tested index for kit C1 and C8 stand out downwardly from the rest of the extraction kits and are often subjected to a much higher error rate than the other tested kits. Also, in the case of C6.1 and C6.2 kits, we can observe differences in the index's values, although they are characterized with a similar amount and quality of the extracted DNA. Kits C2 and C5, have the highest values of all the indexes tested, which could suggest obtaining the most complex microbial population with a high number of species, that can in fact represent the closest assessment to the actual microbial structure of this particular soil sample. Disproportion in the index values, especially for two kits: C1 and C8, and their high error rates, may represent a structure of the microbial population that significantly differs from the actual composition of that population.

Generally, soil DNA extraction protocols can be divided into the two main types of extraction, direct and indirect (Daniel, 2005). It is implied that the indirect methods can yield DNA from 20% to 50% of microorganisms present, while direct methods, from even more than 60% (Bakken & Lindahl, 1995; Robe et al., 2003). It was presumed that with the use of direct extraction methods, the isolated DNA better represents the microbial population structure, as those methods do not include cell separation of microorganisms from soil matrix. Thus, DNA of microorganisms that adhere to the soil particles is included in the population structure analysis (Daniel, 2005). The C1 kit extraction procedure is similar to indirect methods and this might be one of the reasons why this kit differs from other kits. However, the C4 kit protocol for DNA extraction is also similar to indirect methods, but it does not deviate so substantially, as C1, from the other kits. However, in the study performed by Courtois et al. (2001), in order to compare the DNA directly extracted from the soil, with the DNA isolated from cells separated from the soil matrix, there were no significant differences found in the spectrum of bacterial diversity. Nevertheless, Courtois et al. (2001) used for their analysis a different approach, also based on the 16S rRNA gene, than in the study presented here. Their results were accurate for a soil sample which was tested in their study and for applied by them extraction protocols. It is worth mentioning that the exact composition of the soil sample may influence the performance of a given kit, and some kits which are particularly effective for one type of sample may fail when extracting DNA from other samples.

Rarefication analysis of the obtained data revealed trends indicating that sampling of microbial communities is close to being complete for each analyzed kit, which can also indicate the final efficiency of a particular extraction kit. Rarefication analyses are similar for kits C2, C3, C5, C6.1, and C6.2, with low level of error rate among replicates. Rarefication curve for C8 and C1 significantly depart from the other curves, and in addition the C8 curve has a high value of error rates. Also, principal coordinate analysis at the phylum level showed that microbial community composition created with the use of the C1 kit is significantly different from other tested kits. This analysis revealed a large variation between replicates of the C8 kit. The microbial community structures created for the C4 kit, as well as the C1 kit based on an indirect method, stand out from the other tested kits, which are also perceived when considering the diversity indexes. Taken together, in order to receive good quality and reproducible data, several conditions must be fulfilled: (1) a relatively large amount of good quality DNA must be obtained, enabling enzymatic reactions and metagenomics sequencing; (2) a large amount of good quality reads must be obtained, which yields the same population structure between the extraction replicates; (3) high values of diversity indexes, and (4) low values of error rate between the extraction replicates.

When tracking changes in microbial composition over time, between environments, with respect to seasonal and ecological changes, reproducibility of extraction kit replicates are of high importance not only in the aspect of the amount of DNA obtained, but particularly due to the diversity of the obtained material (Morgan et al., 2010). This can be crucial when considering harsh and extreme conditions of quickly changing environments (Handelsman et al., 2007). Using an appropriate extraction kit with listed above features can provide good quality material, suitable for comparative analysis not only within one project, but also for comparisons between studies.

In some studies, the main goal is to catalog all the organisms present. In that case, Morgan et al. (2010) suggested to use multiple DNA extraction procedures for a single environment sample in order to increase the likelihood of discovering every organism in the tested sample. This strategy should be also used in research focused on finding new genes encoding proteins or genes involved in resistance to antibiotics or in pollutant degradation. Morgan et al. (2010) also concluded that representation of microbial species in a given sample can depend not only on the DNA extraction protocol, but also on the organism's growth phase, cloning bias (if used), sequencing efficiency and sequence coverage, as well as the genome copy number. All of those factors can influence the final conclusions about the microbial community structure, so the final decision should be made as to what is the dominant goal of a given study. Our results show that the C1 kit may be preferred in order to establish the representatives of the [Thermi] phylum or *Firmicutes* in a particular soil sample, but due to lack of reproducibility between the extraction replicates, it may not necessarily reveal the real composition of the microbial community structure. At the same time, some of the less abundant phyla may be omitted in the analyses, as it was in this particular case, when 20 of less abundant phyla were absent when using the C1 kit.

Generally speaking, microbial structure of the analyzed soil sample indicated three phyla: *Proteobacteria*,* Acidobacteria*, and *Actinobacteria*, to be the most abundant in the population. Their contribution to the structure differs among the extraction protocols. For two kits, C1 and C5, the *Acidobacteria* are dominant, while for the rest of the tested kits, *Proteobacteria* were dominant, with their share amounting to over 65% (C8). At the high level of taxonomic ranks, this shows how a microbial community structure can differ depending on the DNA extraction method used. Statistical analysis indicated that abundance of different phyla in the microbial structure of 24 out of 32 recognized phyla is significantly different when using various extraction protocols. These differences are seen among the less common phyla as well as the most abundant ones. Following through to the lower taxonomic ranks, we can observe further differences in the abundance of bacteria between the extraction protocols. Significant differences in bacterial structure of human fecal samples were also found when using different extraction protocols (Wesolowska‐Andersen et al., 2014). Also, some of the extraction methods used in that study estimated a lower abundance of certain genera.

Soil samples can be quite problematic not only due to the variety of enzymatic inhibitors that can be co‐extracted with DNA, but also because of mineral particles of different size and organic compounds present at various stages of decomposition (Daniel, 2005). These issues can be solved only by experimental testing. The study presented here allows to compare certain features of the extracted DNA, and more importantly, the final microbial population structure, based on the DNA recovered with the use of different extraction kits. Although in this particular case, the C1 or C8 kits seem to significantly stand out from other tested kits, their utility for different soil samples should be considered and experimentally verified for each sample.

It is important to indicate that the soil sample used in this study was very unusual. The sampling area became a landfill of phosphogypsum post‐production wastes. Currently, this area is subjected to reclamation, and was covered with sludge from wastewater treatment plants, which is characterized by the presence of heavy metals, such as copper, zinc, cadmium, lead, chromium, and nickel; the area is also covered with a vegetation. It resulted in unusual composition of various heavy metals and mineral compounds. Thus, the results obtained in current study should be treated with caution, when other type of soil is to be investigated. As tested kits might be optimized using different type of soil samples, the kits which showed low performance in our study may show excellent DNA extraction efficiency when used on less complex samples.

Based on those facts, we propose that for a single soil sample, as well as for other heterogeneous environmental samples, many DNA extraction kits should be tested at an early stage of the study. Especially, as it was already shown during several tests, that extraction protocols can influence the conclusions about the structure of a pure bacterial culture, when conditions are homogenous (Morgan et al., 2010). Later, based on the amount and quality of the obtained DNA, and also on diversity of the sequences and diversity indexes, as well as reproducibility of the kit extractions, the most suitable protocol should be selected for further analysis. In some cases, in order to improve the analysis, the tests and comparisons of various DNA extraction protocols should be pursued throughout the whole analysis, not only at the early stage of establishing the amount and quality of the recovered DNA. However, this involves an increase in the project costs and is much more time consuming, but when selecting a particular DNA extraction method, one should be aware of its limitations and alternatives. Nonetheless, it could improve the data analysis and thus could be a useful approach in order to present the closest assessment to the actual structure of the microbial population. However, it should be noted that in such studies the view of the final microbial community structure also depends on the sequencing technology and bioinformatics tools used, which were beyond consideration in this study.

## Conflict of Interest

None declared.
