# Supplementary material for: The choice of the DNA extraction method may influence the outcome of the soil microbial community structure analysis
Source: Microbiologyopen. 2017 Feb 20;6(4):e00453. doi: 10.1002/mbo3.453 (PMC5552907; doi:10.1002/mbo3.453)
Supplement: Supplementary file 1 [file MBO3-6-na-s001.pdf]

**Supplementary Table 1.** User evaluation of the DNA extraction kits according to the convenience of their use, the time spent on extraction and cost per sample. Ranked in the range from 1 to 8. 1 – the best in the category, 8 – the last in the category.

| **Kit ID** | **Extraction type** | **Time-consuming** | **Userfriendly** | **Cost (per sample)** | **Avarege mark** | **Final place** |
|---|---|---|---|---|---|---|
| C1 | Indirect | 7 | 8 | 1 | 5.33 | 7 |
| **C2** | **Direct** | **1** | **1** | **6** | **2.67** | **1** |
| **C3** | **Direct** | **2** | **2** | **4** | **2.67** | **1** |
| C4 | Indirect | 8 | 7 | 8 | 7.67 | 8 |
| C5 | Direct | 3 | 3 | 7 | 4.33 | 4 |
| C6 | Direct | 5 | 5 | 5 | 5.00 | 5 |
| **C7** | **Direct** | **4** | **4** | **2** | **3.33** | **3** |
| C8 | Direct | 6 | 6 | 3 | 5.00 | 5 |
