# Supplementary material for: The choice of the DNA extraction method may influence the outcome of the soil microbial community structure analysis
Source: Microbiologyopen. 2017 Feb 20;6(4):e00453. doi: 10.1002/mbo3.453 (PMC5552907; doi:10.1002/mbo3.453)
Supplement: Supplementary file 2 [file MBO3-6-na-s002.pdf]

Supplementary Table 2. Detailed taxonomic analyses at different ranks with data obtained with the tested kits. The table shows the relative abundance of microbial 16S rDNA sequences for each tested kit, at different taxonomic levels.

Kingdom	Phylum	Class	Order	Familly	Genus	C1	C2	C3	C4	C5	C6	C7	C8	C6.1	C6.2
Archaea	Crenarchaeota	Thaumarchaeota	Nitrososphaerales	Nitrososphaeraceae	Candidatus Nitrososphaera	0,00%	0,03%	0,01%	0,01%	0,01%	0,00%	0,00%	0,00%	0,00%	0,01%
Archaea	Euryarchaeota	Methanobacteria	Methanobacteriales	Methanobacteriaceae	Methanobacterium	0,00%	0,00%	0,00%	0,00%	0,00%	0,00%	0,00%	0,01%	0,00%	0,00%
Archaea	Euryarchaeota	Methanomicrobia	Methanosarcinales	Methanosarcinaceae	Methanosarcina	0,00%	0,00%	0,00%	0,00%	0,00%	0,00%	0,00%	0,00%	0,00%	0,00%
Bacteria	AD3	ABS-6				0,00%	0,01%	0,00%	0,01%	0,00%	0,00%	0,00%	0,00%	0,00%	0,00%
Bacteria	Acidobacteria	Acidobacteria-5				0,00%	0,03%	0,02%	0,01%	0,02%	0,02%	0,02%	0,00%	0,02%	0,02%
Bacteria	Acidobacteria	Acidobacteria-6	CCU21			0,00%	0,03%	0,02%	0,02%	0,04%	0,02%	0,03%	0,01%	0,02%	0,03%
Bacteria	Acidobacteria	Acidobacteria-6	iii1-15			0,00%	1,31%	1,10%	1,52%	1,54%	1,14%	0,96%	0,97%	1,03%	1,28%
Bacteria	Acidobacteria	Acidobacteria-6	iii1-15	RB40		0,00%	0,11%	0,12%	0,20%	0,12%	0,14%	0,09%	0,06%	0,11%	0,17%
Bacteria	Acidobacteria	Acidobacteria-6	iii1-15	mb2424		0,00%	0,04%	0,04%	0,21%	0,04%	0,04%	0,04%	0,15%	0,03%	0,06%
Bacteria	Acidobacteria	Acidobacteriia	Acidobacteriales	Acidobacteriaceae		0,00%	3,28%	3,77%	0,49%	3,26%	3,79%	3,67%	0,66%	3,49%	4,15%
Bacteria	Acidobacteria	Acidobacteriia	Acidobacteriales	Acidobacteriaceae	Edaphobacter	0,00%	0,10%	0,20%	0,04%	0,12%	0,17%	0,21%	0,08%	0,16%	0,18%
Bacteria	Acidobacteria	Acidobacteriia	Acidobacteriales	Acidobacteriaceae	Terriglobus	0,00%	0,02%	0,01%	0,00%	0,01%	0,01%	0,01%	0,00%	0,02%	0,01%
Bacteria	Acidobacteria	Acidobacteriia	Acidobacteriales	Koribacteraceae		0,00%	1,39%	1,35%	0,20%	1,37%	1,42%	1,12%	0,28%	1,42%	1,42%
Bacteria	Acidobacteria	Acidobacteriia	Acidobacteriales	Koribacteraceae	Candidatus Koribacter	0,00%	0,17%	0,17%	0,03%	0,19%	0,19%	0,19%	0,00%	0,18%	0,19%
Bacteria	Acidobacteria	DA052	Ellin6513			0,00%	0,20%	0,17%	0,01%	0,18%	0,21%	0,19%	0,02%	0,22%	0,20%
Bacteria	Acidobacteria	EC1113				0,00%	0,00%	0,00%	0,00%	0,00%	0,00%	0,00%	0,00%	0,00%	0,00%
Bacteria	Acidobacteria	Solibacteres	Solibacterales			0,00%	1,25%	1,21%	0,20%	1,37%	1,42%	1,36%	0,19%	1,35%	1,51%
Bacteria	Acidobacteria	Solibacteres	Solibacterales	Solibacteraceae		0,00%	0,70%	1,21%	0,10%	0,94%	0,78%	0,60%	0,23%	0,68%	0,91%
Bacteria	Acidobacteria	Solibacteres	Solibacterales	Solibacteraceae	Candidatus Solibacter	0,00%	0,87%	0,93%	0,22%	0,93%	0,89%	0,77%	0,15%	0,81%	0,99%
Bacteria	Acidobacteria	Solibacteres	Solibacterales	[Bryobacteraceae]		0,00%	0,00%	0,00%	0,00%	0,00%	0,00%	0,00%	0,00%	0,00%	0,00%
Bacteria	Acidobacteria	Sva0725	Sva0725			0,00%	0,00%	0,00%	0,00%	0,01%	0,00%	0,00%	0,00%	0,00%	0,01%
Bacteria	Acidobacteria	TM1				0,00%	0,01%	0,01%	0,00%	0,01%	0,01%	0,01%	0,01%	0,01%	0,00%
Bacteria	Acidobacteria	[Chloracidobacteria]	42332			0,00%	0,00%	0,00%	0,00%	0,00%	0,00%	0,00%	0,00%	0,00%	0,00%
Bacteria	Acidobacteria	[Chloracidobacteria]	DS-100			0,00%	0,01%	0,01%	0,02%	0,01%	0,01%	0,01%	0,00%	0,01%	0,01%
Bacteria	Acidobacteria	[Chloracidobacteria]	PK29			0,00%	0,00%	0,00%	0,00%	0,00%	0,00%	0,00%	0,00%	0,00%	0,00%
Bacteria	Acidobacteria	[Chloracidobacteria]	RB41			0,00%	0,35%	0,15%	0,13%	0,27%	0,27%	0,28%	0,03%	0,24%	0,30%
Bacteria	Acidobacteria	[Chloracidobacteria]	RB41	Ellin6075		0,00%	0,51%	0,28%	0,24%	0,42%	0,49%	0,65%	0,03%	0,46%	0,52%
Bacteria	Acidobacteria	iii1-8	32-20			0,00%	0,06%	0,04%	0,01%	0,05%	0,05%	0,07%	0,00%	0,05%	0,05%
Bacteria	Acidobacteria	iii1-8	DS-18			0,00%	0,29%	0,11%	0,20%	0,28%	0,26%	0,46%	0,03%	0,25%	0,28%
Bacteria	Acidobacteria	iii1-8	SJA-36			0,00%	0,00%	0,00%	0,00%	0,00%	0,00%	0,00%	0,00%	0,00%	0,00%
Bacteria	Actinobacteria	Acidimicrobiia	Acidimicrobiales	Other		0,00%	0,02%	0,00%	0,00%	0,02%	0,00%	0,01%	0,00%	0,01%	0,00%
Bacteria	Actinobacteria	Acidimicrobiia	Acidimicrobiales			0,00%	2,67%	1,65%	2,89%	2,58%	2,24%	1,27%	0,41%	2,21%	2,28%
Bacteria	Actinobacteria	Acidimicrobiia	Acidimicrobiales	AKIW874		0,00%	0,00%	0,00%	0,00%	0,00%	0,00%	0,00%	0,01%	0,00%	0,00%
Bacteria	Actinobacteria	Acidimicrobiia	Acidimicrobiales	C111		0,00%	0,54%	0,46%	0,18%	0,55%	0,39%	0,23%	0,06%	0,38%	0,41%
Bacteria	Actinobacteria	Acidimicrobiia	Acidimicrobiales	EB1017		0,00%	0,64%	0,80%	0,16%	0,74%	0,43%	0,37%	0,15%	0,38%	0,48%
Bacteria	Actinobacteria	Acidimicrobiia	Acidimicrobiales	Iamiaceae	Iamia	0,00%	0,32%	0,24%	0,06%	0,30%	0,35%	0,13%	0,04%	0,37%	0,34%
Bacteria	Actinobacteria	Actinobacteria	Actinomycetales	Other		0,00%	0,49%	0,37%	0,17%	0,49%	0,32%	0,24%	0,05%	0,31%	0,34%
Bacteria	Actinobacteria	Actinobacteria	Actinomycetales			0,00%	1,29%	1,29%	0,42%	1,52%	0,95%	0,63%	0,20%	0,91%	1,00%
Bacteria	Actinobacteria	Actinobacteria	Actinomycetales	Actinomycetaceae	Actinomyces	0,00%	0,00%	0,00%	0,00%	0,00%	0,00%	0,00%	0,00%	0,00%	0,00%
Bacteria	Actinobacteria	Actinobacteria	Actinomycetales	Actinomycetaceae	N09	0,00%	0,00%	0,00%	0,24%	0,00%	0,00%	0,00%	1,15%	0,00%	0,00%
Bacteria	Actinobacteria	Actinobacteria	Actinomycetales	Actinospicaceae		0,00%	0,05%	0,02%	0,00%	0,05%	0,02%	0,07%	0,01%	0,01%	0,02%
Bacteria	Actinobacteria	Actinobacteria	Actinomycetales	Actinosynnemataceae		0,00%	0,04%	0,05%	0,00%	0,06%	0,02%	0,02%	0,03%	0,02%	0,02%
Bacteria	Actinobacteria	Actinobacteria	Actinomycetales	Brevibacteriaceae	Brevibacterium	0,11%	0,00%	0,00%	0,00%	0,00%	0,00%	0,00%	0,00%	0,00%	0,00%
Bacteria	Actinobacteria	Actinobacteria	Actinomycetales	Cellulomonadaceae	Actinotalea	0,00%	0,00%	0,00%	0,01%	0,00%	0,00%	0,00%	0,01%	0,00%	0,00%
Bacteria	Actinobacteria	Actinobacteria	Actinomycetales	Cellulomonadaceae	Cellulomonas	0,00%	0,00%	0,01%	0,00%	0,01%	0,00%	0,01%	0,03%	0,00%	0,00%
Bacteria	Actinobacteria	Actinobacteria	Actinomycetales	Corynebacteriaceae	Corynebacterium	4,90%	0,00%	0,00%	0,20%	0,00%	0,03%	0,01%	0,40%	0,00%	0,05%
Bacteria	Actinobacteria	Actinobacteria	Actinomycetales	Cryptosporangiaceae		0,00%	0,00%	0,00%	0,00%	0,00%	0,00%	0,00%	0,00%	0,00%	0,00%
Bacteria	Actinobacteria	Actinobacteria	Actinomycetales	Dermabacteraceae	Brachybacterium	0,00%	0,00%	0,00%	0,00%	0,00%	0,00%	0,00%	0,01%	0,00%	0,00%
Bacteria	Actinobacteria	Actinobacteria	Actinomycetales	Dermacoccaceae	Dermacoccus	0,00%	0,01%	0,01%	0,01%	0,01%	0,01%	0,00%	0,00%	0,01%	0,01%

Bacteria	Actinobacteria	Actinobacteria	Actinomycetales	Dietziaceae		0,77%	0,00%	0,00%	0,00%	0,00%	0,00%	0,00%	0,03%	0,00%	0,00%
Bacteria	Actinobacteria	Actinobacteria	Actinomycetales	Dietziaceae	Dietzia	0,00%	0,00%	0,00%	0,00%	0,00%	0,00%	0,00%	0,00%	0,00%	0,00%
Bacteria	Actinobacteria	Actinobacteria	Actinomycetales	Frankiaceae		0,00%	1,45%	1,34%	0,39%	1,55%	0,76%	0,57%	0,16%	0,79%	0,72%
Bacteria	Actinobacteria	Actinobacteria	Actinomycetales	Frankiaceae	Frankia	0,00%	0,01%	0,01%	0,00%	0,01%	0,00%	0,00%	0,00%	0,00%	0,00%
Bacteria	Actinobacteria	Actinobacteria	Actinomycetales	Geodermatophilaceae		0,00%	0,32%	0,25%	0,04%	0,27%	0,26%	0,17%	0,10%	0,26%	0,26%
Bacteria	Actinobacteria	Actinobacteria	Actinomycetales	Geodermatophilaceae		0,00%	0,09%	0,08%	0,01%	0,09%	0,05%	0,04%	0,02%	0,05%	0,06%
Bacteria	Actinobacteria	Actinobacteria	Actinomycetales	Geodermatophilaceae	Geodermatophilus	0,00%	0,00%	0,00%	0,00%	0,00%	0,00%	0,00%	0,01%	0,00%	0,00%
Bacteria	Actinobacteria	Actinobacteria	Actinomycetales	Geodermatophilaceae	Modestobacter	0,00%	0,03%	0,03%	0,00%	0,03%	0,02%	0,01%	0,01%	0,02%	0,02%
Bacteria	Actinobacteria	Actinobacteria	Actinomycetales	Intrasporangiaceae		0,00%	0,56%	0,62%	1,41%	0,72%	0,28%	0,19%	0,13%	0,28%	0,28%
Bacteria	Actinobacteria	Actinobacteria	Actinomycetales	Intrasporangiaceae		0,00%	0,12%	0,12%	0,17%	0,17%	0,06%	0,04%	0,03%	0,06%	0,06%
Bacteria	Actinobacteria	Actinobacteria	Actinomycetales	Intrasporangiaceae	Phycicoccus	0,00%	0,01%	0,01%	0,01%	0,01%	0,00%	0,00%	0,00%	0,00%	0,01%
Bacteria	Actinobacteria	Actinobacteria	Actinomycetales	Intrasporangiaceae	Terracoccus	0,00%	0,02%	0,04%	0,01%	0,03%	0,02%	0,01%	0,00%	0,02%	0,02%
Bacteria	Actinobacteria	Actinobacteria	Actinomycetales	Kineosporiaceae		0,00%	0,06%	0,07%	0,03%	0,07%	0,03%	0,02%	0,01%	0,02%	0,03%
Bacteria	Actinobacteria	Actinobacteria	Actinomycetales	Kineosporiaceae		0,00%	0,11%	0,28%	0,01%	0,15%	0,08%	0,24%	0,09%	0,07%	0,08%
Bacteria	Actinobacteria	Actinobacteria	Actinomycetales	Kineosporiaceae	Kineococcus	0,00%	0,01%	0,01%	0,00%	0,00%	0,00%	0,00%	0,02%	0,00%	0,00%
Bacteria	Actinobacteria	Actinobacteria	Actinomycetales	Microbacteriaceae		0,00%	0,07%	0,09%	0,06%	0,08%	0,05%	0,04%	0,05%	0,05%	0,05%
Bacteria	Actinobacteria	Actinobacteria	Actinomycetales	Microbacteriaceae		0,00%	0,48%	0,80%	0,40%	0,65%	0,27%	0,31%	0,11%	0,28%	0,26%
Bacteria	Actinobacteria	Actinobacteria	Actinomycetales	Microbacteriaceae	Cryocola	0,40%	0,02%	0,02%	0,12%	0,03%	0,01%	0,02%	0,30%	0,01%	0,01%
Bacteria	Actinobacteria	Actinobacteria	Actinomycetales	Microbacteriaceae	Microbacterium	0,00%	0,00%	0,01%	0,01%	0,00%	0,00%	0,00%	0,03%	0,00%	0,01%
Bacteria	Actinobacteria	Actinobacteria	Actinomycetales	Microbacteriaceae	Rathayibacter	0,00%	0,01%	0,01%	0,01%	0,02%	0,01%	0,01%	0,01%	0,01%	0,01%
Bacteria	Actinobacteria	Actinobacteria	Actinomycetales	Microbacteriaceae	Salinibacterium	0,01%	0,53%	0,53%	0,33%	0,54%	0,38%	0,43%	0,20%	0,38%	0,37%
Bacteria	Actinobacteria	Actinobacteria	Actinomycetales	Micrococcaceae		0,00%	0,01%	0,01%	0,00%	0,04%	0,00%	0,00%	0,00%	0,01%	0,00%
Bacteria	Actinobacteria	Actinobacteria	Actinomycetales	Micrococcaceae		0,01%	0,68%	0,68%	0,53%	0,89%	0,38%	0,16%	0,15%	0,39%	0,37%
Bacteria	Actinobacteria	Actinobacteria	Actinomycetales	Micrococcaceae	Arthrobacter	0,13%	0,71%	0,60%	0,44%	0,78%	0,37%	0,18%	0,17%	0,39%	0,35%
Bacteria	Actinobacteria	Actinobacteria	Actinomycetales	Micrococcaceae	Kocuria	0,01%	0,00%	0,00%	0,00%	0,00%	0,00%	0,00%	0,00%	0,00%	0,00%
Bacteria	Actinobacteria	Actinobacteria	Actinomycetales	Micrococcaceae	Micrococcus	1,18%	0,00%	0,00%	0,01%	0,00%	0,00%	0,00%	0,03%	0,00%	0,00%
Bacteria	Actinobacteria	Actinobacteria	Actinomycetales	Micromonosporaceae		0,00%	0,25%	0,42%	0,01%	0,32%	0,16%	0,22%	0,12%	0,15%	0,17%
Bacteria	Actinobacteria	Actinobacteria	Actinomycetales	Micromonosporaceae		0,00%	1,09%	2,27%	0,04%	1,69%	0,76%	1,22%	0,41%	0,78%	0,72%
Bacteria	Actinobacteria	Actinobacteria	Actinomycetales	Micromonosporaceae	Actinoplanes	0,00%	0,05%	0,11%	0,00%	0,09%	0,04%	0,11%	0,04%	0,03%	0,05%
Bacteria	Actinobacteria	Actinobacteria	Actinomycetales	Micromonosporaceae	Catellatospora	0,00%	0,00%	0,00%	0,00%	0,00%	0,00%	0,00%	0,00%	0,00%	0,00%
Bacteria	Actinobacteria	Actinobacteria	Actinomycetales	Micromonosporaceae	Dactylosporangium	0,00%	0,12%	0,33%	0,01%	0,32%	0,13%	0,18%	0,08%	0,12%	0,13%
Bacteria	Actinobacteria	Actinobacteria	Actinomycetales	Micromonosporaceae	Micromonospora	0,00%	0,00%	0,00%	0,00%	0,00%	0,00%	0,00%	0,00%	0,00%	0,00%
Bacteria	Actinobacteria	Actinobacteria	Actinomycetales	Micromonosporaceae	Pilimelia	0,00%	0,01%	0,01%	0,00%	0,01%	0,01%	0,01%	0,00%	0,00%	0,01%
Bacteria	Actinobacteria	Actinobacteria	Actinomycetales	Micromonosporaceae	Virgisporangium	0,00%	0,04%	0,05%	0,00%	0,04%	0,02%	0,04%	0,02%	0,03%	0,02%
Bacteria	Actinobacteria	Actinobacteria	Actinomycetales	Mycobacteriaceae	Mycobacterium	0,00%	1,79%	2,41%	0,07%	2,43%	0,84%	1,52%	0,50%	0,89%	0,77%
Bacteria	Actinobacteria	Actinobacteria	Actinomycetales	Nakamurellaceae		0,00%	0,32%	0,25%	0,00%	0,32%	0,18%	0,09%	0,04%	0,17%	0,19%
Bacteria	Actinobacteria	Actinobacteria	Actinomycetales	Nocardiaceae		0,00%	0,00%	0,00%	0,00%	0,00%	0,00%	0,00%	0,00%	0,00%	0,00%
Bacteria	Actinobacteria	Actinobacteria	Actinomycetales	Nocardiaceae		0,00%	0,02%	0,03%	0,00%	0,02%	0,01%	0,02%	0,01%	0,01%	0,01%
Bacteria	Actinobacteria	Actinobacteria	Actinomycetales	Nocardiaceae	Nocardia	0,00%	0,04%	0,03%	0,01%	0,02%	0,02%	0,01%	0,00%	0,02%	0,02%
Bacteria	Actinobacteria	Actinobacteria	Actinomycetales	Nocardiaceae	Rhodococcus	0,00%	0,07%	0,09%	0,02%	0,07%	0,03%	0,02%	0,00%	0,03%	0,03%
Bacteria	Actinobacteria	Actinobacteria	Actinomycetales	Nocardioidaceae		0,00%	1,74%	2,64%	3,21%	2,25%	1,00%	0,80%	0,86%	1,02%	0,98%
Bacteria	Actinobacteria	Actinobacteria	Actinomycetales	Nocardioidaceae	Actinopolymorpha	0,00%	0,00%	0,01%	0,00%	0,00%	0,00%	0,00%	0,00%	0,00%	0,00%
Bacteria	Actinobacteria	Actinobacteria	Actinomycetales	Nocardioidaceae	Aeromicrobium	0,00%	0,17%	0,54%	0,07%	0,20%	0,11%	0,16%	0,01%	0,11%	0,12%
Bacteria	Actinobacteria	Actinobacteria	Actinomycetales	Nocardioidaceae	Friedmanniella	0,00%	0,06%	0,08%	0,00%	0,04%	0,05%	0,04%	0,03%	0,06%	0,04%
Bacteria	Actinobacteria	Actinobacteria	Actinomycetales	Nocardioidaceae	Kribbella	0,00%	0,33%	0,40%	0,04%	0,37%	0,20%	0,19%	0,04%	0,19%	0,22%
Bacteria	Actinobacteria	Actinobacteria	Actinomycetales	Nocardioidaceae	Nocardioides	0,00%	1,09%	1,38%	0,51%	1,39%	0,51%	0,45%	0,35%	0,53%	0,49%
Bacteria	Actinobacteria	Actinobacteria	Actinomycetales	Nocardioidaceae	Pimelobacter	0,00%	0,00%	0,01%	0,00%	0,00%	0,00%	0,00%	0,00%	0,00%	0,00%
Bacteria	Actinobacteria	Actinobacteria	Actinomycetales	Nocardioidaceae	Propionicimonas	0,00%	0,00%	0,01%	0,00%	0,00%	0,00%	0,00%	0,00%	0,00%	0,00%
Bacteria	Actinobacteria	Actinobacteria	Actinomycetales	Promicromonosporaceae	Promicromonospora	0,00%	0,00%	0,00%	0,00%	0,00%	0,00%	0,00%	0,02%	0,00%	0,00%
Bacteria	Actinobacteria	Actinobacteria	Actinomycetales	Promicromonosporaceae	Xylanimicrobium	0,00%	0,00%	0,00%	0,01%	0,00%	0,00%	0,00%	0,03%	0,00%	0,00%
Bacteria	Actinobacteria	Actinobacteria	Actinomycetales	Propionibacteriaceae		0,00%	0,06%	0,08%	0,00%	0,04%	0,05%	0,03%	0,06%	0,05%	0,06%
Bacteria	Actinobacteria	Actinobacteria	Actinomycetales	Propionibacteriaceae	Microlunatus	0,00%	0,04%	0,03%	0,00%	0,02%	0,02%	0,01%	0,00%	0,02%	0,02%
Bacteria	Actinobacteria	Actinobacteria	Actinomycetales	Propionibacteriaceae	Propionibacterium	32,59%	0,00%	0,00%	0,04%	0,00%	0,00%	0,01%	0,31%	0,00%	0,00%

Bacteria	Actinobacteria	Actinobacteria	Actinomycetales	Pseudonocardiaceae		0,00%	0,00%	0,00%	0,00%	0,00%	0,00%	0,00%	0,00%	0,00%	0,00%
Bacteria	Actinobacteria	Actinobacteria	Actinomycetales	Pseudonocardiaceae		0,00%	0,03%	0,01%	0,00%	0,04%	0,01%	0,01%	0,02%	0,01%	0,02%
Bacteria	Actinobacteria	Actinobacteria	Actinomycetales	Pseudonocardiaceae	Actinomycetospora	0,00%	0,04%	0,04%	0,00%	0,07%	0,02%	0,03%	0,02%	0,02%	0,02%
Bacteria	Actinobacteria	Actinobacteria	Actinomycetales	Pseudonocardiaceae	Amycolatopsis	0,00%	0,04%	0,12%	0,03%	0,07%	0,04%	0,16%	0,04%	0,04%	0,03%
Bacteria	Actinobacteria	Actinobacteria	Actinomycetales	Pseudonocardiaceae	Prauserella	0,00%	0,00%	0,00%	0,00%	0,00%	0,00%	0,00%	0,02%	0,00%	0,00%
Bacteria	Actinobacteria	Actinobacteria	Actinomycetales	Pseudonocardiaceae	Pseudonocardia	0,00%	0,14%	0,11%	0,00%	0,15%	0,08%	0,09%	0,01%	0,07%	0,08%
Bacteria	Actinobacteria	Actinobacteria	Actinomycetales	Pseudonocardiaceae	Saccharopolyspora	0,00%	0,00%	0,00%	0,00%	0,00%	0,00%	0,00%	0,00%	0,00%	0,00%
Bacteria	Actinobacteria	Actinobacteria	Actinomycetales	Sporichthyaceae		0,00%	0,46%	0,32%	0,31%	0,43%	0,32%	0,27%	0,05%	0,36%	0,27%
Bacteria	Actinobacteria	Actinobacteria	Actinomycetales	Sporichthyaceae	Sporichthya	0,00%	0,02%	0,01%	0,00%	0,02%	0,01%	0,01%	0,00%	0,01%	0,01%
Bacteria	Actinobacteria	Actinobacteria	Actinomycetales	Streptomycetaceae		0,00%	0,09%	0,11%	0,01%	0,10%	0,04%	0,04%	0,01%	0,04%	0,04%
Bacteria	Actinobacteria	Actinobacteria	Actinomycetales	Streptomycetaceae		0,00%	0,33%	0,37%	0,01%	0,42%	0,09%	0,21%	0,03%	0,09%	0,09%
Bacteria	Actinobacteria	Actinobacteria	Actinomycetales	Streptomycetaceae	Streptacidiphilus	0,00%	0,01%	0,01%	0,00%	0,01%	0,00%	0,00%	0,00%	0,00%	0,00%
Bacteria	Actinobacteria	Actinobacteria	Actinomycetales	Streptomycetaceae	Streptomyces	0,00%	2,00%	1,87%	0,05%	2,77%	0,76%	1,13%	0,84%	0,85%	0,64%
Bacteria	Actinobacteria	Actinobacteria	Actinomycetales	Streptosporangiaceae		0,00%	0,02%	0,02%	0,00%	0,02%	0,01%	0,00%	0,00%	0,01%	0,01%
Bacteria	Actinobacteria	Actinobacteria	Actinomycetales	Streptosporangiaceae	Nonomuraea	0,00%	0,03%	0,03%	0,00%	0,04%	0,01%	0,01%	0,01%	0,01%	0,01%
Bacteria	Actinobacteria	Actinobacteria	Actinomycetales	Streptosporangiaceae	Sphaerisporangium	0,00%	0,02%	0,02%	0,00%	0,03%	0,01%	0,00%	0,00%	0,01%	0,01%
Bacteria	Actinobacteria	Actinobacteria	Actinomycetales	Streptosporangiaceae	Streptosporangium	0,00%	0,06%	0,07%	0,00%	0,08%	0,02%	0,01%	0,01%	0,02%	0,02%
Bacteria	Actinobacteria	Actinobacteria	Actinomycetales	Thermomonosporaceae		0,00%	0,01%	0,01%	0,00%	0,02%	0,00%	0,01%	0,00%	0,00%	0,00%
Bacteria	Actinobacteria	Actinobacteria	Actinomycetales	Thermomonosporaceae	Actinoallomurus	0,00%	0,03%	0,04%	0,00%	0,05%	0,01%	0,01%	0,00%	0,01%	0,01%
Bacteria	Actinobacteria	Actinobacteria	Actinomycetales	Thermomonosporaceae	Actinocorallia	0,00%	0,02%	0,01%	0,00%	0,01%	0,01%	0,02%	0,00%	0,01%	0,01%
Bacteria	Actinobacteria	Actinobacteria	Actinomycetales	Thermomonosporaceae	Actinomadura	0,00%	0,04%	0,06%	0,00%	0,08%	0,02%	0,01%	0,01%	0,02%	0,02%
Bacteria	Actinobacteria	Actinobacteria	Actinomycetales	Williamsiaceae	Williamsia	0,00%	0,09%	0,05%	0,00%	0,12%	0,08%	0,05%	0,00%	0,10%	0,05%
Bacteria	Actinobacteria	Actinobacteria	Bifidobacteriales	Bifidobacteriaceae		0,00%	0,00%	0,00%	0,00%	0,00%	0,00%	0,00%	0,00%	0,00%	0,00%
Bacteria	Actinobacteria	Actinobacteria	Bifidobacteriales	Bifidobacteriaceae	Bifidobacterium	0,00%	0,00%	0,00%	0,03%	0,00%	0,00%	0,00%	0,03%	0,00%	0,00%
Bacteria	Actinobacteria	Actinobacteria	Micrococcales			0,00%	0,00%	0,00%	0,00%	0,00%	0,00%	0,00%	0,00%	0,00%	0,00%
Bacteria	Actinobacteria	MB-A2-108				0,00%	0,46%	0,24%	0,11%	0,38%	0,51%	0,20%	0,02%	0,49%	0,54%
Bacteria	Actinobacteria	MB-A2-108	0319-7L14			0,00%	0,08%	0,04%	0,00%	0,07%	0,03%	0,01%	0,00%	0,03%	0,03%
Bacteria	Actinobacteria	Rubrobacteria	Rubrobacterales	Rubrobacteraceae	Rubrobacter	2,80%	0,00%	0,00%	0,01%	0,00%	0,00%	0,00%	0,07%	0,00%	0,00%
Bacteria	Actinobacteria	Thermoleophilia	Gaiellales			0,00%	0,51%	0,43%	0,10%	0,50%	0,26%	0,18%	0,12%	0,22%	0,31%
Bacteria	Actinobacteria	Thermoleophilia	Gaiellales	AK1AB1	02E	0,00%	0,03%	0,02%	0,11%	0,03%	0,02%	0,01%	0,02%	0,02%	0,01%
Bacteria	Actinobacteria	Thermoleophilia	Gaiellales	Gaiellaceae		0,00%	3,43%	2,42%	9,73%	3,48%	2,02%	1,36%	2,17%	2,15%	1,86%
Bacteria	Actinobacteria	Thermoleophilia	Solirubrobacterales			0,00%	2,25%	2,68%	0,50%	2,54%	1,12%	1,07%	0,59%	1,09%	1,16%
Bacteria	Actinobacteria	Thermoleophilia	Solirubrobacterales	Conexibacteraceae		0,00%	0,26%	0,32%	0,09%	0,33%	0,15%	0,13%	0,12%	0,15%	0,15%
Bacteria	Actinobacteria	Thermoleophilia	Solirubrobacterales	Conexibacteraceae	Conexibacter	0,00%	0,03%	0,03%	0,02%	0,04%	0,03%	0,02%	0,01%	0,03%	0,03%
Bacteria	Actinobacteria	Thermoleophilia	Solirubrobacterales	Patulibacteraceae		0,00%	0,70%	0,59%	0,69%	0,78%	0,28%	0,16%	0,13%	0,31%	0,25%
Bacteria	Actinobacteria	Thermoleophilia	Solirubrobacterales	Patulibacteraceae	Patulibacter	0,00%	0,00%	0,00%	0,00%	0,00%	0,00%	0,00%	0,00%	0,00%	0,00%
Bacteria	Actinobacteria	Thermoleophilia	Solirubrobacterales	Solirubrobacteraceae		0,00%	0,82%	0,87%	0,36%	0,85%	0,44%	0,41%	0,32%	0,42%	0,46%
Bacteria	Actinobacteria	Thermoleophilia	Solirubrobacterales	Solirubrobacteraceae	Solirubrobacter	0,00%	0,08%	0,08%	0,03%	0,13%	0,05%	0,05%	0,06%	0,06%	0,04%
Bacteria	Armatimonadetes	0319-6E2				0,00%	0,01%	0,00%	0,01%	0,01%	0,01%	0,01%	0,01%	0,01%	0,01%
Bacteria	Armatimonadetes	Armatimonadia	Armatimonadales	Armatimonadaceae		0,00%	0,01%	0,02%	0,01%	0,01%	0,01%	0,03%	0,02%	0,01%	0,01%
Bacteria	Armatimonadetes	Armatimonadia	FW68			0,00%	0,01%	0,01%	0,00%	0,00%	0,01%	0,02%	0,02%	0,01%	0,01%
Bacteria	Armatimonadetes	Chthonomonadetes	Chthonomonadales	Chthonomonadaceae		0,00%	0,00%	0,00%	0,00%	0,00%	0,00%	0,01%	0,00%	0,00%	0,00%
Bacteria	Armatimonadetes	Chthonomonadetes	SJA-22			0,00%	0,00%	0,00%	0,00%	0,00%	0,00%	0,00%	0,00%	0,00%	0,00%
Bacteria	Armatimonadetes	OPB50				0,00%	0,00%	0,00%	0,00%	0,00%	0,00%	0,00%	0,00%	0,00%	0,00%
Bacteria	Armatimonadetes	SJA-176				0,00%	0,00%	0,00%	0,00%	0,00%	0,00%	0,00%	0,00%	0,00%	0,00%
Bacteria	Armatimonadetes	SJA-176	TP122			0,00%	0,00%	0,00%	0,00%	0,00%	0,00%	0,00%	0,00%	0,00%	0,00%
Bacteria	Armatimonadetes	[Fimbriimonadia]	[Fimbriimonadales]	[Fimbriimonadaceae]		0,00%	0,01%	0,00%	0,00%	0,00%	0,01%	0,01%	0,00%	0,01%	0,00%
Bacteria	Armatimonadetes	[Fimbriimonadia]	[Fimbriimonadales]	[Fimbriimonadaceae]	Fimbriimonas	0,00%	0,22%	0,08%	0,05%	0,15%	0,20%	0,14%	0,03%	0,18%	0,22%
Bacteria	BRC1	PRR-11				0,00%	0,00%	0,00%	0,00%	0,00%	0,00%	0,00%	0,00%	0,00%	0,00%
Bacteria	Bacteroidetes	Bacteroidia	Bacteroidales			0,00%	0,00%	0,00%	0,00%	0,00%	0,00%	0,00%	0,00%	0,00%	0,00%
Bacteria	Bacteroidetes	Bacteroidia	Bacteroidales	Porphyromonadaceae		0,00%	0,00%	0,00%	0,01%	0,00%	0,00%	0,00%	0,06%	0,00%	0,00%
Bacteria	Bacteroidetes	Bacteroidia	Bacteroidales	Prevotellaceae	Prevotella	0,84%	0,00%	0,00%	0,28%	0,00%	0,00%	0,00%	0,60%	0,00%	0,00%
Bacteria	Bacteroidetes	Cytophagia	Cytophagales			0,00%	0,00%	0,00%	0,00%	0,00%	0,00%	0,00%	0,00%	0,00%	0,00%

Bacteria	Bacteroidetes	Cytophagia	Cytophagales	Cytophagaceae		0,00%	0,06%	0,11%	0,01%	0,05%	0,05%	0,14%	0,02%	0,06%	0,04%
Bacteria	Bacteroidetes	Cytophagia	Cytophagales	Cytophagaceae	Cytophaga	0,00%	0,02%	0,05%	0,00%	0,01%	0,03%	0,21%	0,02%	0,04%	0,02%
Bacteria	Bacteroidetes	Cytophagia	Cytophagales	Cytophagaceae	Dyadobacter	0,00%	0,01%	0,04%	0,00%	0,00%	0,00%	0,00%	0,00%	0,01%	0,00%
Bacteria	Bacteroidetes	Cytophagia	Cytophagales	Cytophagaceae	Hymenobacter	0,00%	0,02%	0,04%	0,00%	0,02%	0,03%	0,04%	0,00%	0,03%	0,03%
Bacteria	Bacteroidetes	Cytophagia	Cytophagales	Cytophagaceae	Spirosoma	0,00%	0,00%	0,01%	0,00%	0,00%	0,00%	0,00%	0,00%	0,00%	0,00%
Bacteria	Bacteroidetes	Cytophagia	Cytophagales	Cytophagaceae	Sporocytophaga	0,00%	0,01%	0,01%	0,00%	0,00%	0,01%	0,01%	0,01%	0,01%	0,01%
Bacteria	Bacteroidetes	Cytophagia	Cytophagales	[Amoebophilaceae]	Candidatus Amoebophilus	0,00%	0,00%	0,00%	0,00%	0,00%	0,00%	0,00%	0,00%	0,00%	0,00%
Bacteria	Bacteroidetes	Flavobacteriia	Flavobacteriales	Cryomorphaceae		0,00%	0,01%	0,02%	0,01%	0,01%	0,01%	0,02%	0,00%	0,02%	0,01%
Bacteria	Bacteroidetes	Flavobacteriia	Flavobacteriales	Cryomorphaceae	Fluviicola	0,00%	0,01%	0,05%	0,00%	0,01%	0,04%	0,12%	0,00%	0,05%	0,02%
Bacteria	Bacteroidetes	Flavobacteriia	Flavobacteriales	Flavobacteriaceae		0,00%	0,00%	0,00%	0,00%	0,00%	0,00%	0,00%	0,00%	0,00%	0,00%
Bacteria	Bacteroidetes	Flavobacteriia	Flavobacteriales	Flavobacteriaceae	Flavobacterium	0,00%	0,03%	0,05%	0,05%	0,02%	0,04%	0,74%	0,00%	0,05%	0,02%
Bacteria	Bacteroidetes	Flavobacteriia	Flavobacteriales	[Weeksellaceae]	Chryseobacterium	0,00%	0,00%	0,00%	0,03%	0,00%	0,01%	0,02%	0,00%	0,01%	0,00%
Bacteria	Bacteroidetes	Flavobacteriia	Flavobacteriales	[Weeksellaceae]	Cloacibacterium	0,00%	0,00%	0,00%	0,00%	0,00%	0,00%	0,00%	0,04%	0,00%	0,00%
Bacteria	Bacteroidetes	Flavobacteriia	Flavobacteriales	[Weeksellaceae]	Wautersiella	0,00%	0,00%	0,00%	0,00%	0,00%	0,00%	0,00%	0,00%	0,00%	0,00%
Bacteria	Bacteroidetes	Sphingobacteriia	Sphingobacteriales			0,00%	0,14%	0,13%	0,03%	0,08%	0,12%	0,31%	0,04%	0,13%	0,10%
Bacteria	Bacteroidetes	Sphingobacteriia	Sphingobacteriales	Sphingobacteriaceae		0,00%	0,58%	0,65%	0,95%	0,36%	0,78%	2,02%	0,78%	0,80%	0,74%
Bacteria	Bacteroidetes	Sphingobacteriia	Sphingobacteriales	Sphingobacteriaceae	Olivibacter	0,00%	0,00%	0,00%	0,00%	0,00%	0,00%	0,00%	0,00%	0,00%	0,00%
Bacteria	Bacteroidetes	Sphingobacteriia	Sphingobacteriales	Sphingobacteriaceae	Pedobacter	0,00%	0,02%	0,04%	0,17%	0,01%	0,03%	0,07%	0,01%	0,03%	0,03%
Bacteria	Bacteroidetes	Sphingobacteriia	Sphingobacteriales	Sphingobacteriaceae	Sphingobacterium	0,00%	0,00%	0,00%	0,00%	0,00%	0,00%	0,00%	0,01%	0,00%	0,00%
Bacteria	Bacteroidetes	[Rhodothermi]	[Rhodothermales]	Rhodothermaceae	Rubricoccus	0,00%	0,00%	0,00%	0,00%	0,00%	0,00%	0,00%	0,00%	0,00%	0,00%
Bacteria	Bacteroidetes	[Saprospirae]	[Saprospirales]			0,00%	0,01%	0,01%	0,00%	0,01%	0,01%	0,01%	0,00%	0,01%	0,00%
Bacteria	Bacteroidetes	[Saprospirae]	[Saprospirales]	Chitinophagaceae		0,00%	1,11%	1,63%	0,58%	0,92%	1,50%	2,68%	0,59%	1,53%	1,46%
Bacteria	Bacteroidetes	[Saprospirae]	[Saprospirales]	Chitinophagaceae	Chitinophaga	0,00%	0,02%	0,01%	0,02%	0,01%	0,01%	0,03%	0,01%	0,01%	0,01%
Bacteria	Bacteroidetes	[Saprospirae]	[Saprospirales]	Chitinophagaceae	Flavisolibacter	0,00%	0,15%	0,16%	0,04%	0,12%	0,20%	0,37%	0,02%	0,20%	0,20%
Bacteria	Bacteroidetes	[Saprospirae]	[Saprospirales]	Chitinophagaceae	Niabella	0,00%	0,00%	0,00%	0,00%	0,00%	0,00%	0,00%	0,00%	0,00%	0,00%
Bacteria	Bacteroidetes	[Saprospirae]	[Saprospirales]	Chitinophagaceae	Sediminibacterium	0,00%	0,00%	0,00%	0,02%	0,00%	0,01%	0,02%	0,01%	0,01%	0,00%
Bacteria	Bacteroidetes	[Saprospirae]	[Saprospirales]	Chitinophagaceae	Segetibacter	0,00%	0,04%	0,04%	0,01%	0,02%	0,06%	0,05%	0,01%	0,05%	0,06%
Bacteria	Bacteroidetes	[Saprospirae]	[Saprospirales]	Saprospiraceae		0,00%	0,02%	0,02%	0,01%	0,02%	0,02%	0,04%	0,01%	0,02%	0,02%
Bacteria	Chlamydiae	Chlamydiia	Chlamydiales	Criblamydiaceae		0,00%	0,01%	0,00%	0,00%	0,00%	0,00%	0,00%	0,00%	0,00%	0,00%
Bacteria	Chlorobi					0,00%	0,04%	0,01%	0,01%	0,01%	0,02%	0,03%	0,00%	0,02%	0,02%
Bacteria	Chlorobi	BSV26	PK329			0,00%	0,00%	0,00%	0,00%	0,00%	0,00%	0,00%	0,00%	0,00%	0,00%
Bacteria	Chlorobi	OPB56				0,00%	0,00%	0,00%	0,00%	0,00%	0,00%	0,00%	0,01%	0,00%	0,00%
Bacteria	Chlorobi	SJA-28				0,00%	0,00%	0,00%	0,00%	0,00%	0,00%	0,00%	0,00%	0,00%	0,00%
Bacteria	Chloroflexi					0,00%	0,00%	0,00%	0,00%	0,00%	0,00%	0,00%	0,00%	0,00%	0,00%
Bacteria	Chloroflexi	Anaerolineae	A31	S47		0,00%	0,10%	0,03%	0,01%	0,05%	0,02%	0,01%	0,00%	0,02%	0,02%
Bacteria	Chloroflexi	Anaerolineae	CFB-26			0,00%	0,00%	0,00%	0,00%	0,00%	0,00%	0,00%	0,00%	0,00%	0,00%
Bacteria	Chloroflexi	Anaerolineae	Caldilineales	Caldilineaceae		0,00%	0,07%	0,06%	0,01%	0,05%	0,03%	0,02%	0,01%	0,03%	0,03%
Bacteria	Chloroflexi	Anaerolineae	H39			0,00%	0,10%	0,04%	0,02%	0,06%	0,05%	0,04%	0,01%	0,04%	0,05%
Bacteria	Chloroflexi	Anaerolineae	SBR1031	A4b		0,00%	0,20%	0,18%	0,04%	0,10%	0,12%	0,19%	0,04%	0,12%	0,12%
Bacteria	Chloroflexi	Anaerolineae	SBR1031	oc28		0,00%	0,01%	0,00%	0,00%	0,01%	0,00%	0,00%	0,00%	0,00%	0,00%
Bacteria	Chloroflexi	Anaerolineae	envOPS12			0,00%	0,00%	0,00%	0,00%	0,00%	0,00%	0,00%	0,00%	0,00%	0,00%
Bacteria	Chloroflexi	C0119				0,00%	0,16%	0,16%	0,06%	0,15%	0,11%	0,08%	0,02%	0,11%	0,10%
Bacteria	Chloroflexi	Chloroflexi	Herpetosiphonales			0,00%	0,00%	0,00%	0,00%	0,00%	0,00%	0,00%	0,00%	0,00%	0,00%
Bacteria	Chloroflexi	Chloroflexi	[Roseiflexales]	[Kouleothrixaceae]		0,00%	0,27%	0,27%	0,01%	0,27%	0,14%	0,19%	0,02%	0,14%	0,13%
Bacteria	Chloroflexi	Ellin6529				1,51%	4,78%	2,81%	1,17%	4,26%	2,76%	1,53%	0,77%	2,77%	2,74%
Bacteria	Chloroflexi	Gitt-GS-136				0,00%	1,23%	1,01%	0,65%	1,22%	0,68%	0,29%	0,18%	0,59%	0,80%
Bacteria	Chloroflexi	Ktedonobacteria	JG30-KF-AS9			0,00%	0,01%	0,03%	0,00%	0,00%	0,01%	0,01%	0,00%	0,01%	0,00%
Bacteria	Chloroflexi	P2-11E				0,00%	0,04%	0,02%	0,06%	0,04%	0,05%	0,03%	0,06%	0,06%	0,05%
Bacteria	Chloroflexi	S085				0,00%	0,31%	0,17%	0,06%	0,26%	0,14%	0,11%	0,04%	0,13%	0,15%
Bacteria	Chloroflexi	TK10				0,00%	0,03%	0,02%	0,00%	0,03%	0,02%	0,01%	0,00%	0,02%	0,02%
Bacteria	Chloroflexi	TK10	AKYG885	5B-12		0,00%	0,03%	0,01%	0,00%	0,03%	0,02%	0,01%	0,00%	0,02%	0,03%
Bacteria	Chloroflexi	TK10	AKYG885	Dolo		0,00%	0,18%	0,08%	0,00%	0,14%	0,05%	0,04%	0,05%	0,05%	0,06%
Bacteria	Chloroflexi	TK10	B07	WMSP1		0,00%	0,04%	0,02%	0,01%	0,04%	0,01%	0,01%	0,02%	0,01%	0,01%

Bacteria	Chloroflexi	TK10	B07	WMSP1	FFCH4570	0,00%	0,74%	0,46%	0,13%	0,61%	0,35%	0,25%	0,11%	0,34%	0,37%
Bacteria	Chloroflexi	TK17				0,00%	0,14%	0,06%	0,07%	0,13%	0,06%	0,02%	0,02%	0,06%	0,06%
Bacteria	Chloroflexi	TK17	mle1-48			0,00%	0,07%	0,03%	0,02%	0,06%	0,03%	0,03%	0,00%	0,03%	0,04%
Bacteria	Chloroflexi	Thermomicrobia				0,00%	0,10%	0,06%	0,00%	0,07%	0,06%	0,01%	0,05%	0,04%	0,08%
Bacteria	Chloroflexi	Thermomicrobia	Ellin6537			0,00%	0,33%	0,19%	0,02%	0,22%	0,12%	0,04%	0,00%	0,12%	0,11%
Bacteria	Chloroflexi	Thermomicrobia	JG30-KF-CM45			0,00%	0,50%	0,36%	0,01%	0,53%	0,15%	0,07%	0,05%	0,13%	0,16%
Bacteria	Cyanobacteria					0,00%	0,01%	0,00%	0,00%	0,01%	0,01%	0,01%	0,00%	0,01%	0,01%
Bacteria	Cyanobacteria	4C0d-2	MLE1-12			0,00%	0,01%	0,00%	0,14%	0,01%	0,00%	0,01%	0,00%	0,01%	0,00%
Bacteria	Cyanobacteria	4C0d-2	SM1D11			0,00%	0,02%	0,02%	0,01%	0,01%	0,02%	0,01%	0,03%	0,02%	0,02%
Bacteria	Cyanobacteria	Chloroplast				0,00%	0,00%	0,00%	0,00%	0,00%	0,00%	0,00%	0,00%	0,00%	0,00%
Bacteria	Cyanobacteria	Chloroplast	Chlorophyta			0,00%	0,05%	0,06%	0,00%	0,02%	0,03%	0,02%	0,04%	0,03%	0,03%
Bacteria	Cyanobacteria	Chloroplast	Chlorophyta	Trebouxiophyceae		0,00%	0,03%	0,02%	0,00%	0,02%	0,02%	0,01%	0,00%	0,01%	0,02%
Bacteria	Cyanobacteria	Chloroplast	Chlorophyta	Trebouxiophyceae		0,00%	0,03%	0,04%	0,00%	0,01%	0,01%	0,01%	0,00%	0,01%	0,01%
Bacteria	Cyanobacteria	Chloroplast	Chlorophyta	Trebouxiophyceae	Coccomyxa	0,00%	0,02%	0,01%	0,00%	0,01%	0,01%	0,01%	0,00%	0,01%	0,01%
Bacteria	Cyanobacteria	Chloroplast	Stramenopiles			0,00%	0,07%	0,07%	0,00%	0,03%	0,05%	0,03%	0,00%	0,05%	0,06%
Bacteria	Cyanobacteria	Chloroplast	Streptophyta			0,32%	0,47%	0,67%	0,03%	0,13%	0,29%	0,17%	0,20%	0,36%	0,20%
Bacteria	Cyanobacteria	ML635J-21				0,00%	0,09%	0,04%	0,02%	0,06%	0,05%	0,03%	0,00%	0,06%	0,05%
Bacteria	Cyanobacteria	Nostocophycideae	Nostocales	Nostocaceae		0,00%	0,00%	0,00%	0,00%	0,00%	0,00%	0,00%	0,00%	0,00%	0,00%
Bacteria	Cyanobacteria	Nostocophycideae	Nostocales	Nostocaceae		0,00%	0,00%	0,00%	0,00%	0,00%	0,00%	0,00%	0,00%	0,00%	0,00%
Bacteria	Cyanobacteria	Nostocophycideae	Nostocales	Nostocaceae	Nostoc	0,00%	0,05%	0,01%	0,00%	0,08%	0,02%	0,01%	0,00%	0,02%	0,03%
Bacteria	Cyanobacteria	Oscillatoriophycideae	Oscillatoriales	Phormidiaceae	Phormidium	0,00%	0,00%	0,00%	0,00%	0,00%	0,00%	0,00%	0,00%	0,00%	0,00%
Bacteria	Cyanobacteria	Synechococcophycideae	Pseudanabaenales	Pseudanabaenaceae	Leptolyngbya	0,00%	0,02%	0,00%	0,00%	0,01%	0,01%	0,00%	0,00%	0,00%	0,01%
Bacteria	Elusimicrobia	Elusimicrobia	Elusimicrobiales			0,00%	0,02%	0,00%	0,00%	0,01%	0,01%	0,01%	0,00%	0,01%	0,02%
Bacteria	Elusimicrobia	Elusimicrobia	FAC88			0,00%	0,00%	0,00%	0,00%	0,00%	0,00%	0,00%	0,00%	0,00%	0,00%
Bacteria	Elusimicrobia	Elusimicrobia	IIb			0,00%	0,01%	0,00%	0,00%	0,01%	0,01%	0,01%	0,00%	0,01%	0,00%
Bacteria	FBP					0,00%	0,02%	0,02%	0,03%	0,02%	0,02%	0,03%	0,00%	0,02%	0,02%
Bacteria	FCPU426					0,00%	0,00%	0,00%	0,00%	0,00%	0,00%	0,00%	0,00%	0,00%	0,00%
Bacteria	Fibrobacteres	Fibrobacteria	258ds10			0,00%	0,02%	0,01%	0,01%	0,02%	0,01%	0,03%	0,01%	0,01%	0,01%
Bacteria	Firmicutes	Bacilli	Bacillales	Other		0,00%	0,20%	1,06%	0,01%	0,63%	0,70%	0,10%	0,10%	0,65%	0,77%
Bacteria	Firmicutes	Bacilli	Bacillales			0,00%	0,00%	0,02%	0,01%	0,01%	0,01%	0,00%	0,00%	0,01%	0,02%
Bacteria	Firmicutes	Bacilli	Bacillales	Alicyclobacillaceae	Alicyclobacillus	0,00%	0,06%	0,25%	0,01%	0,20%	0,13%	0,07%	0,00%	0,13%	0,14%
Bacteria	Firmicutes	Bacilli	Bacillales	Bacillaceae		0,00%	0,00%	0,00%	0,01%	0,00%	0,00%	0,00%	0,00%	0,00%	0,00%
Bacteria	Firmicutes	Bacilli	Bacillales	Bacillaceae		0,00%	0,00%	0,07%	0,01%	0,03%	0,03%	0,02%	0,00%	0,03%	0,04%
Bacteria	Firmicutes	Bacilli	Bacillales	Bacillaceae	Anoxybacillus	0,00%	0,00%	0,00%	0,01%	0,00%	0,00%	0,00%	0,16%	0,00%	0,00%
Bacteria	Firmicutes	Bacilli	Bacillales	Bacillaceae	Bacillus	0,00%	0,39%	2,24%	0,20%	1,22%	1,42%	0,24%	0,16%	1,29%	1,57%
Bacteria	Firmicutes	Bacilli	Bacillales	Bacillaceae	Geobacillus	0,07%	0,00%	0,00%	0,00%	0,00%	0,00%	0,00%	0,00%	0,00%	0,00%
Bacteria	Firmicutes	Bacilli	Bacillales	Bacillaceae	Marinibacillus	0,00%	0,00%	0,00%	0,00%	0,00%	0,00%	0,00%	0,00%	0,00%	0,00%
Bacteria	Firmicutes	Bacilli	Bacillales	Bacillaceae	Virgibacillus	0,00%	0,00%	0,00%	0,00%	0,00%	0,00%	0,00%	0,00%	0,00%	0,00%
Bacteria	Firmicutes	Bacilli	Bacillales	Paenibacillaceae		0,00%	0,00%	0,00%	0,00%	0,00%	0,00%	0,00%	0,00%	0,00%	0,00%
Bacteria	Firmicutes	Bacilli	Bacillales	Paenibacillaceae	Ammoniphilus	0,00%	0,01%	0,02%	0,00%	0,01%	0,01%	0,00%	0,01%	0,01%	0,01%
Bacteria	Firmicutes	Bacilli	Bacillales	Paenibacillaceae	Brevibacillus	0,00%	0,00%	0,01%	0,00%	0,01%	0,01%	0,00%	0,01%	0,01%	0,01%
Bacteria	Firmicutes	Bacilli	Bacillales	Paenibacillaceae	Cohnella	0,00%	0,00%	0,02%	0,00%	0,01%	0,01%	0,00%	0,00%	0,01%	0,02%
Bacteria	Firmicutes	Bacilli	Bacillales	Paenibacillaceae	Paenibacillus	0,00%	0,08%	0,36%	0,01%	0,32%	0,20%	0,09%	0,03%	0,20%	0,20%
Bacteria	Firmicutes	Bacilli	Bacillales	Pasteuriaceae	Pasteuria	0,00%	0,00%	0,00%	0,00%	0,00%	0,00%	0,00%	0,00%	0,00%	0,00%
Bacteria	Firmicutes	Bacilli	Bacillales	Planococcaceae		0,00%	0,00%	0,01%	0,00%	0,01%	0,00%	0,00%	0,03%	0,00%	0,01%
Bacteria	Firmicutes	Bacilli	Bacillales	Planococcaceae		0,00%	0,03%	0,15%	0,03%	0,08%	0,10%	0,02%	0,03%	0,09%	0,10%
Bacteria	Firmicutes	Bacilli	Bacillales	Planococcaceae	Lysinibacillus	0,00%	0,00%	0,02%	0,00%	0,01%	0,01%	0,01%	0,01%	0,01%	0,02%
Bacteria	Firmicutes	Bacilli	Bacillales	Planococcaceae	Paenisporosarcina	0,00%	0,00%	0,01%	0,00%	0,01%	0,01%	0,00%	0,00%	0,01%	0,01%
Bacteria	Firmicutes	Bacilli	Bacillales	Planococcaceae	Planomicrobium	0,00%	0,00%	0,00%	0,00%	0,00%	0,00%	0,00%	0,06%	0,00%	0,00%
Bacteria	Firmicutes	Bacilli	Bacillales	Planococcaceae	Rummeliibacillus	0,00%	0,00%	0,00%	0,00%	0,00%	0,00%	0,00%	0,00%	0,00%	0,00%
Bacteria	Firmicutes	Bacilli	Bacillales	Planococcaceae	Solibacillus	0,00%	0,01%	0,04%	0,00%	0,03%	0,03%	0,00%	0,01%	0,03%	0,03%
Bacteria	Firmicutes	Bacilli	Bacillales	Planococcaceae	Sporosarcina	0,00%	0,06%	0,21%	0,00%	0,15%	0,12%	0,02%	0,06%	0,11%	0,14%
Bacteria	Firmicutes	Bacilli	Bacillales	Sporolactobacillaceae		0,00%	0,00%	0,00%	0,00%	0,00%	0,00%	0,00%	0,00%	0,00%	0,00%

Bacteria	Firmicutes	Bacilli	Bacillales	Staphylococcaceae	Macrococcus	0,00%	0,00%	0,00%	0,00%	0,00%	0,00%	0,00%	0,01%	0,00%	0,00%
Bacteria	Firmicutes	Bacilli	Bacillales	Staphylococcaceae	Staphylococcus	4,91%	0,00%	0,01%	0,04%	0,00%	0,00%	0,01%	0,17%	0,00%	0,00%
Bacteria	Firmicutes	Bacilli	Bacillales	Thermoactinomycetaceae		0,00%	0,00%	0,00%	0,00%	0,00%	0,00%	0,00%	0,00%	0,00%	0,00%
Bacteria	Firmicutes	Bacilli	Bacillales	Thermoactinomycetaceae		0,00%	0,01%	0,01%	0,02%	0,02%	0,01%	0,01%	0,00%	0,01%	0,01%
Bacteria	Firmicutes	Bacilli	Bacillales	Thermoactinomycetaceae	Shimazuella	0,00%	0,00%	0,01%	0,00%	0,01%	0,00%	0,00%	0,00%	0,00%	0,00%
Bacteria	Firmicutes	Bacilli	Bacillales	[Exiguobacteraceae]	Exiguobacterium	0,00%	0,00%	0,00%	0,00%	0,00%	0,00%	0,00%	0,00%	0,00%	0,00%
Bacteria	Firmicutes	Bacilli	Lactobacillales	Aerococcaceae		0,00%	0,00%	0,00%	0,01%	0,00%	0,00%	0,00%	0,03%	0,00%	0,00%
Bacteria	Firmicutes	Bacilli	Lactobacillales	Aerococcaceae	Aerococcus	0,20%	0,00%	0,00%	0,01%	0,00%	0,00%	0,00%	0,00%	0,00%	0,00%
Bacteria	Firmicutes	Bacilli	Lactobacillales	Aerococcaceae	Alkalibacterium	0,00%	0,00%	0,00%	0,01%	0,00%	0,00%	0,00%	0,00%	0,00%	0,00%
Bacteria	Firmicutes	Bacilli	Lactobacillales	Enterococcaceae	Enterococcus	0,01%	0,00%	0,00%	0,00%	0,00%	0,00%	0,00%	0,00%	0,00%	0,00%
Bacteria	Firmicutes	Bacilli	Lactobacillales	Lactobacillaceae	Lactobacillus	0,42%	0,00%	0,00%	0,02%	0,00%	0,00%	0,00%	0,76%	0,00%	0,00%
Bacteria	Firmicutes	Bacilli	Lactobacillales	Leuconostocaceae	Leuconostoc	0,19%	0,00%	0,00%	0,00%	0,00%	0,00%	0,00%	0,05%	0,00%	0,00%
Bacteria	Firmicutes	Bacilli	Lactobacillales	Streptococcaceae	Lactococcus	0,00%	0,00%	0,00%	0,00%	0,00%	0,00%	0,00%	1,48%	0,00%	0,00%
Bacteria	Firmicutes	Bacilli	Lactobacillales	Streptococcaceae	Streptococcus	7,26%	0,00%	0,00%	0,01%	0,00%	0,00%	0,00%	5,04%	0,00%	0,00%
Bacteria	Firmicutes	Bacilli	Turicibacterales	Turicibacteraceae	Turicibacter	0,00%	0,00%	0,01%	0,00%	0,01%	0,01%	0,00%	0,00%	0,01%	0,01%
Bacteria	Firmicutes	Clostridia				0,00%	0,00%	0,00%	0,00%	0,00%	0,00%	0,00%	0,00%	0,00%	0,00%
Bacteria	Firmicutes	Clostridia	Clostridiales			0,00%	0,00%	0,00%	0,00%	0,00%	0,00%	0,00%	0,00%	0,00%	0,00%
Bacteria	Firmicutes	Clostridia	Clostridiales	Christensenellaceae		0,00%	0,00%	0,00%	0,00%	0,00%	0,00%	0,00%	0,00%	0,00%	0,00%
Bacteria	Firmicutes	Clostridia	Clostridiales	Clostridiaceae		0,00%	0,01%	0,07%	0,00%	0,03%	0,04%	0,03%	0,00%	0,04%	0,04%
Bacteria	Firmicutes	Clostridia	Clostridiales	Clostridiaceae		0,00%	0,01%	0,04%	0,00%	0,02%	0,03%	0,01%	0,00%	0,02%	0,04%
Bacteria	Firmicutes	Clostridia	Clostridiales	Clostridiaceae	Alkaliphilus	0,00%	0,00%	0,00%	0,00%	0,00%	0,00%	0,00%	0,00%	0,00%	0,00%
Bacteria	Firmicutes	Clostridia	Clostridiales	Clostridiaceae	Caloramator	0,00%	0,00%	0,00%	0,00%	0,00%	0,00%	0,00%	0,00%	0,00%	0,00%
Bacteria	Firmicutes	Clostridia	Clostridiales	Clostridiaceae	Clostridium	0,00%	0,04%	0,11%	0,00%	0,08%	0,05%	0,02%	0,02%	0,05%	0,05%
Bacteria	Firmicutes	Clostridia	Clostridiales	Clostridiaceae	Oxobacter	0,00%	0,00%	0,00%	0,00%	0,00%	0,00%	0,00%	0,00%	0,00%	0,00%
Bacteria	Firmicutes	Clostridia	Clostridiales	Clostridiaceae	SMB53	0,00%	0,00%	0,00%	0,00%	0,00%	0,00%	0,00%	0,00%	0,00%	0,00%
Bacteria	Firmicutes	Clostridia	Clostridiales	Gracilibacteraceae		0,00%	0,00%	0,01%	0,00%	0,00%	0,00%	0,00%	0,00%	0,00%	0,00%
Bacteria	Firmicutes	Clostridia	Clostridiales	Gracilibacteraceae	Gracilibacter	0,00%	0,00%	0,00%	0,00%	0,00%	0,00%	0,00%	0,01%	0,00%	0,00%
Bacteria	Firmicutes	Clostridia	Clostridiales	Gracilibacteraceae	Lutispora	0,00%	0,00%	0,00%	0,00%	0,00%	0,00%	0,00%	0,00%	0,00%	0,00%
Bacteria	Firmicutes	Clostridia	Clostridiales	Lachnospiraceae		0,00%	0,00%	0,00%	0,01%	0,00%	0,00%	0,00%	0,00%	0,00%	0,00%
Bacteria	Firmicutes	Clostridia	Clostridiales	Lachnospiraceae	Coprococcus	0,00%	0,00%	0,00%	0,00%	0,00%	0,00%	0,00%	0,00%	0,00%	0,00%
Bacteria	Firmicutes	Clostridia	Clostridiales	Lachnospiraceae	Epulopiscium	0,00%	0,00%	0,00%	0,00%	0,00%	0,00%	0,00%	0,00%	0,00%	0,00%
Bacteria	Firmicutes	Clostridia	Clostridiales	Peptococcaceae		0,00%	0,00%	0,00%	0,00%	0,00%	0,00%	0,00%	0,00%	0,00%	0,00%
Bacteria	Firmicutes	Clostridia	Clostridiales	Peptococcaceae		0,00%	0,00%	0,00%	0,00%	0,00%	0,00%	0,00%	0,00%	0,00%	0,00%
Bacteria	Firmicutes	Clostridia	Clostridiales	Peptococcaceae	Desulfosporosinus	0,00%	0,00%	0,01%	0,00%	0,01%	0,00%	0,00%	0,00%	0,00%	0,01%
Bacteria	Firmicutes	Clostridia	Clostridiales	Peptococcaceae	Sporotomaculum	0,00%	0,00%	0,00%	0,00%	0,00%	0,00%	0,00%	0,00%	0,00%	0,00%
Bacteria	Firmicutes	Clostridia	Clostridiales	Peptostreptococcaceae		0,00%	0,00%	0,00%	0,00%	0,00%	0,00%	0,00%	0,00%	0,00%	0,00%
Bacteria	Firmicutes	Clostridia	Clostridiales	Peptostreptococcaceae		0,00%	0,00%	0,00%	0,00%	0,00%	0,01%	0,00%	0,00%	0,00%	0,01%
Bacteria	Firmicutes	Clostridia	Clostridiales	Ruminococcaceae		0,00%	0,01%	0,02%	0,04%	0,01%	0,00%	0,00%	0,03%	0,01%	0,00%
Bacteria	Firmicutes	Clostridia	Clostridiales	Ruminococcaceae	Ruminococcus	0,00%	0,00%	0,00%	0,00%	0,00%	0,00%	0,00%	0,00%	0,00%	0,00%
Bacteria	Firmicutes	Clostridia	Clostridiales	Veillonellaceae		0,00%	0,00%	0,00%	0,01%	0,00%	0,00%	0,00%	0,03%	0,00%	0,00%
Bacteria	Firmicutes	Clostridia	Clostridiales	Veillonellaceae	Dialister	0,00%	0,00%	0,00%	0,00%	0,00%	0,00%	0,00%	0,00%	0,00%	0,00%
Bacteria	Firmicutes	Clostridia	Clostridiales	Veillonellaceae	Pelosinus	0,00%	0,00%	0,00%	0,00%	0,00%	0,00%	0,00%	0,00%	0,00%	0,00%
Bacteria	Firmicutes	Clostridia	Clostridiales	Veillonellaceae	Veillonella	0,00%	0,00%	0,00%	0,00%	0,00%	0,00%	0,00%	0,00%	0,00%	0,00%
Bacteria	Firmicutes	Clostridia	Clostridiales	[Mogibacteriaceae]	Anaerovorax	0,00%	0,00%	0,00%	0,00%	0,00%	0,00%	0,00%	0,00%	0,00%	0,00%
Bacteria	Firmicutes	Clostridia	Clostridiales	[Tissierellaceae]		0,00%	0,00%	0,00%	0,00%	0,00%	0,00%	0,00%	0,03%	0,00%	0,00%
Bacteria	Firmicutes	Clostridia	Clostridiales	[Tissierellaceae]	Anaerococcus	0,35%	0,00%	0,00%	0,00%	0,00%	0,00%	0,00%	0,00%	0,00%	0,00%
Bacteria	Firmicutes	Clostridia	Clostridiales	[Tissierellaceae]	Finegoldia	0,00%	0,00%	0,00%	0,01%	0,00%	0,00%	0,00%	0,00%	0,00%	0,00%
Bacteria	Firmicutes	Clostridia	Clostridiales	[Tissierellaceae]	Peptoniphilus	5,84%	0,00%	0,00%	0,03%	0,00%	0,00%	0,00%	0,26%	0,00%	0,00%
Bacteria	Firmicutes	Clostridia	Clostridiales	[Tissierellaceae]	Sedimentibacter	0,00%	0,00%	0,00%	0,00%	0,00%	0,00%	0,00%	0,00%	0,00%	0,00%
Bacteria	Firmicutes	Clostridia	Clostridiales	[Tissierellaceae]	Tepidimicrobium	0,00%	0,00%	0,00%	0,00%	0,00%	0,00%	0,00%	0,00%	0,00%	0,00%
Bacteria	Firmicutes	Clostridia	MBA08			0,00%	0,00%	0,00%	0,00%	0,00%	0,00%	0,00%	0,00%	0,00%	0,00%
Bacteria	Firmicutes	Clostridia	OPB54			0,00%	0,00%	0,01%	0,00%	0,00%	0,00%	0,00%	0,00%	0,00%	0,00%
Bacteria	Firmicutes	Erysipelotrichi	Erysipelotrichales	Erysipelotrichaceae	PSB-M-3	0,00%	0,00%	0,00%	0,00%	0,00%	0,00%	0,00%	0,00%	0,00%	0,00%

Bacteria	Fusobacteria	Fusobacteriia	Fusobacteriales	Fusobacteriaceae	Fusobacterium	0,00%	0,00%	0,00%	0,00%	0,00%	0,00%	0,00%	0,00%	0,00%	0,00%
Bacteria	Gemmatimonadetes	Gemm-1				0,00%	2,34%	1,32%	0,26%	2,27%	2,13%	1,48%	0,24%	1,93%	2,36%
Bacteria	Gemmatimonadetes	Gemm-2				0,00%	0,02%	0,01%	0,01%	0,02%	0,02%	0,01%	0,04%	0,02%	0,02%
Bacteria	Gemmatimonadetes	Gemm-3				0,00%	0,00%	0,00%	0,00%	0,00%	0,00%	0,00%	0,00%	0,00%	0,00%
Bacteria	Gemmatimonadetes	Gemm-5				0,00%	0,15%	0,03%	0,10%	0,10%	0,09%	0,12%	0,32%	0,08%	0,11%
Bacteria	Gemmatimonadetes	Gemmatimonadetes				0,00%	0,21%	0,24%	0,09%	0,30%	0,35%	0,22%	0,04%	0,34%	0,35%
Bacteria	Gemmatimonadetes	Gemmatimonadetes	Ellin5290			0,00%	0,35%	0,18%	0,10%	0,22%	0,25%	0,26%	0,16%	0,23%	0,28%
Bacteria	Gemmatimonadetes	Gemmatimonadetes	Gemmatimonadales			0,00%	0,02%	0,06%	0,01%	0,03%	0,04%	0,03%	0,01%	0,04%	0,04%
Bacteria	Gemmatimonadetes	Gemmatimonadetes	Gemmatimonadales	Ellin5301		0,00%	0,88%	0,62%	0,16%	0,83%	0,75%	0,78%	0,09%	0,67%	0,84%
Bacteria	Gemmatimonadetes	Gemmatimonadetes	Gemmatimonadales	Gemmatimonadaceae	Gemmatimonas	0,00%	0,00%	0,00%	0,00%	0,01%	0,01%	0,00%	0,00%	0,01%	0,00%
Bacteria	Gemmatimonadetes	Gemmatimonadetes	KD8-87			0,00%	0,00%	0,01%	0,00%	0,00%	0,00%	0,00%	0,00%	0,00%	0,00%
Bacteria	Gemmatimonadetes	Gemmatimonadetes	N1423WL			0,00%	1,85%	0,59%	2,07%	1,43%	1,35%	1,43%	2,42%	1,25%	1,47%
Bacteria	MVP-21					0,00%	0,01%	0,01%	0,06%	0,02%	0,01%	0,01%	0,01%	0,01%	0,01%
Bacteria	Nitrospirae	Nitrospira	Nitrospirales	0319-6A21		0,00%	0,01%	0,01%	0,00%	0,01%	0,01%	0,01%	0,00%	0,01%	0,01%
Bacteria	Nitrospirae	Nitrospira	Nitrospirales	Nitrospiraceae		0,00%	0,01%	0,00%	0,00%	0,01%	0,00%	0,00%	0,00%	0,00%	0,01%
Bacteria	Nitrospirae	Nitrospira	Nitrospirales	Nitrospiraceae	JG37-AG-70	0,00%	0,00%	0,00%	0,00%	0,01%	0,00%	0,00%	0,00%	0,00%	0,00%
Bacteria	Nitrospirae	Nitrospira	Nitrospirales	Nitrospiraceae	Nitrospira	0,00%	0,06%	0,05%	0,00%	0,06%	0,06%	0,04%	0,02%	0,06%	0,07%
Bacteria	OD1					0,00%	0,00%	0,00%	0,00%	0,00%	0,00%	0,00%	0,01%	0,00%	0,00%
Bacteria	OD1	SM2F11				0,00%	0,04%	0,02%	0,02%	0,01%	0,02%	0,05%	0,01%	0,02%	0,02%
Bacteria	OD1	ZB2				0,00%	0,04%	0,03%	0,02%	0,02%	0,03%	0,05%	0,02%	0,03%	0,04%
Bacteria	OP11	OP11-1				0,00%	0,00%	0,00%	0,00%	0,00%	0,00%	0,00%	0,00%	0,00%	0,00%
Bacteria	OP11	OP11-3				0,00%	0,00%	0,00%	0,00%	0,00%	0,00%	0,00%	0,00%	0,00%	0,00%
Bacteria	OP11	OP11-4				0,00%	0,00%	0,00%	0,01%	0,00%	0,00%	0,00%	0,01%	0,00%	0,00%
Bacteria	OP11	WCHB1-64				0,00%	0,00%	0,00%	0,00%	0,00%	0,00%	0,00%	0,01%	0,00%	0,00%
Bacteria	OP11	WCHB1-64	d153			0,00%	0,00%	0,00%	0,00%	0,00%	0,00%	0,00%	0,00%	0,00%	0,00%
Bacteria	Planctomycetes	BD7-11				0,00%	0,01%	0,00%	0,00%	0,01%	0,01%	0,00%	0,00%	0,01%	0,01%
Bacteria	Planctomycetes	OM190	agg27			0,00%	0,00%	0,00%	0,00%	0,00%	0,00%	0,00%	0,00%	0,00%	0,00%
Bacteria	Planctomycetes	Phycisphaerae	Phycisphaerales			0,00%	0,00%	0,01%	0,00%	0,01%	0,01%	0,01%	0,00%	0,01%	0,01%
Bacteria	Planctomycetes	Phycisphaerae	WD2101			0,00%	0,87%	0,63%	0,08%	0,68%	0,59%	0,56%	0,10%	0,48%	0,72%
Bacteria	Planctomycetes	Pla4				0,00%	0,00%	0,00%	0,00%	0,00%	0,00%	0,00%	0,00%	0,00%	0,00%
Bacteria	Planctomycetes	Planctomycetia	B97			0,00%	0,00%	0,00%	0,01%	0,00%	0,00%	0,00%	0,00%	0,00%	0,00%
Bacteria	Planctomycetes	Planctomycetia	Gemmatales	Gemmataceae		0,00%	0,46%	0,37%	0,15%	0,34%	0,16%	0,09%	0,05%	0,15%	0,19%
Bacteria	Planctomycetes	Planctomycetia	Gemmatales	Gemmataceae	Gemmata	0,00%	0,24%	0,26%	0,06%	0,21%	0,11%	0,07%	0,05%	0,10%	0,12%
Bacteria	Planctomycetes	Planctomycetia	Gemmatales	Isosphaeraceae		0,00%	0,34%	0,94%	0,06%	0,26%	0,23%	0,14%	0,12%	0,20%	0,27%
Bacteria	Planctomycetes	Planctomycetia	Pirellulales	Pirellulaceae		0,00%	0,21%	0,25%	0,06%	0,15%	0,09%	0,12%	0,07%	0,08%	0,10%
Bacteria	Planctomycetes	Planctomycetia	Pirellulales	Pirellulaceae	A17	0,00%	0,03%	0,03%	0,02%	0,03%	0,02%	0,02%	0,03%	0,01%	0,02%
Bacteria	Planctomycetes	Planctomycetia	Pirellulales	Pirellulaceae	Pirellula	0,00%	0,03%	0,03%	0,01%	0,02%	0,01%	0,01%	0,00%	0,01%	0,01%
Bacteria	Planctomycetes	Planctomycetia	Planctomycetales	Planctomycetaceae	Planctomyces	0,00%	0,10%	0,08%	0,06%	0,06%	0,06%	0,03%	0,05%	0,05%	0,07%
Bacteria	Planctomycetes	vadinHA49	DH61			0,00%	0,02%	0,01%	0,00%	0,01%	0,01%	0,01%	0,01%	0,02%	0,01%
Bacteria	Proteobacteria	Alphaproteobacteria	Other			0,00%	0,00%	0,00%	0,00%	0,00%	0,00%	0,00%	0,00%	0,00%	0,00%
Bacteria	Proteobacteria	Alphaproteobacteria				0,00%	0,06%	0,04%	0,02%	0,05%	0,05%	0,05%	0,04%	0,05%	0,05%
Bacteria	Proteobacteria	Alphaproteobacteria	BD7-3			0,00%	0,01%	0,01%	0,00%	0,01%	0,01%	0,02%	0,00%	0,01%	0,01%
Bacteria	Proteobacteria	Alphaproteobacteria	Caulobacterales	Caulobacteraceae		0,00%	0,00%	0,00%	0,01%	0,00%	0,00%	0,01%	0,00%	0,00%	0,00%
Bacteria	Proteobacteria	Alphaproteobacteria	Caulobacterales	Caulobacteraceae		0,00%	0,28%	0,20%	0,23%	0,28%	0,34%	0,31%	0,15%	0,30%	0,39%
Bacteria	Proteobacteria	Alphaproteobacteria	Caulobacterales	Caulobacteraceae	Asticcacaulis	0,00%	0,02%	0,03%	0,06%	0,03%	0,04%	0,12%	0,04%	0,03%	0,06%
Bacteria	Proteobacteria	Alphaproteobacteria	Caulobacterales	Caulobacteraceae	Brevundimonas	0,00%	0,00%	0,00%	0,00%	0,00%	0,00%	0,00%	0,00%	0,00%	0,00%
Bacteria	Proteobacteria	Alphaproteobacteria	Caulobacterales	Caulobacteraceae	Caulobacter	0,00%	0,01%	0,02%	0,03%	0,03%	0,04%	0,12%	0,02%	0,05%	0,02%
Bacteria	Proteobacteria	Alphaproteobacteria	Caulobacterales	Caulobacteraceae	Mycoplana	0,00%	0,01%	0,00%	0,01%	0,01%	0,01%	0,01%	0,00%	0,01%	0,01%
Bacteria	Proteobacteria	Alphaproteobacteria	Caulobacterales	Caulobacteraceae	Phenylobacterium	0,00%	0,40%	0,37%	0,28%	0,43%	0,56%	0,60%	0,10%	0,52%	0,62%
Bacteria	Proteobacteria	Alphaproteobacteria	Ellin329			0,00%	2,33%	1,70%	1,50%	1,71%	2,56%	2,56%	1,33%	2,63%	2,48%
Bacteria	Proteobacteria	Alphaproteobacteria	Rhizobiales	Other		0,00%	0,04%	0,05%	0,11%	0,04%	0,03%	0,03%	0,01%	0,03%	0,03%
Bacteria	Proteobacteria	Alphaproteobacteria	Rhizobiales			0,00%	0,73%	0,52%	0,53%	0,73%	0,46%	0,41%	0,37%	0,46%	0,45%
Bacteria	Proteobacteria	Alphaproteobacteria	Rhizobiales	Aurantimonadaceae		0,00%	0,00%	0,02%	0,01%	0,00%	0,00%	0,00%	0,01%	0,00%	0,00%

Bacteria	Proteobacteria	Alphaproteobacteria	Rhizobiales	Beijerinckiaceae		0,00%	0,00%	0,00%	0,01%	0,00%	0,00%	0,00%	0,00%	0,00%	0,00%
Bacteria	Proteobacteria	Alphaproteobacteria	Rhizobiales	Beijerinckiaceae		0,00%	0,10%	0,10%	0,07%	0,11%	0,08%	0,05%	0,08%	0,07%	0,08%
Bacteria	Proteobacteria	Alphaproteobacteria	Rhizobiales	Beijerinckiaceae	Beijerinckia	0,00%	0,00%	0,00%	0,01%	0,00%	0,00%	0,00%	0,00%	0,00%	0,00%
Bacteria	Proteobacteria	Alphaproteobacteria	Rhizobiales	Bradyrhizobiaceae		0,00%	0,28%	0,44%	0,60%	0,30%	0,34%	0,28%	0,13%	0,35%	0,32%
Bacteria	Proteobacteria	Alphaproteobacteria	Rhizobiales	Bradyrhizobiaceae		0,17%	1,71%	2,62%	2,10%	1,90%	1,67%	1,49%	0,73%	1,82%	1,50%
Bacteria	Proteobacteria	Alphaproteobacteria	Rhizobiales	Bradyrhizobiaceae	Balneimonas	0,00%	0,02%	0,01%	0,01%	0,01%	0,01%	0,00%	0,00%	0,01%	0,01%
Bacteria	Proteobacteria	Alphaproteobacteria	Rhizobiales	Bradyrhizobiaceae	Bosea	0,00%	0,00%	0,01%	0,01%	0,01%	0,00%	0,00%	0,00%	0,00%	0,00%
Bacteria	Proteobacteria	Alphaproteobacteria	Rhizobiales	Bradyrhizobiaceae	Bradyrhizobium	0,00%	0,04%	0,04%	0,05%	0,03%	0,03%	0,03%	0,00%	0,03%	0,02%
Bacteria	Proteobacteria	Alphaproteobacteria	Rhizobiales	Brucellaceae	Ochrobactrum	0,00%	0,00%	0,00%	0,00%	0,00%	0,00%	0,00%	0,00%	0,00%	0,00%
Bacteria	Proteobacteria	Alphaproteobacteria	Rhizobiales	Hyphomicrobiaceae		0,00%	0,00%	0,00%	0,00%	0,00%	0,00%	0,00%	0,00%	0,00%	0,00%
Bacteria	Proteobacteria	Alphaproteobacteria	Rhizobiales	Hyphomicrobiaceae		0,00%	0,58%	1,24%	0,46%	0,84%	0,27%	0,44%	0,26%	0,22%	0,33%
Bacteria	Proteobacteria	Alphaproteobacteria	Rhizobiales	Hyphomicrobiaceae	Devosia	0,00%	0,83%	0,72%	0,62%	0,92%	0,77%	0,68%	0,25%	0,77%	0,78%
Bacteria	Proteobacteria	Alphaproteobacteria	Rhizobiales	Hyphomicrobiaceae	Hyphomicrobium	0,00%	0,12%	0,14%	0,12%	0,10%	0,05%	0,07%	0,04%	0,05%	0,05%
Bacteria	Proteobacteria	Alphaproteobacteria	Rhizobiales	Hyphomicrobiaceae	Parvibaculum	0,00%	0,03%	0,01%	0,01%	0,02%	0,05%	0,03%	0,01%	0,04%	0,06%
Bacteria	Proteobacteria	Alphaproteobacteria	Rhizobiales	Hyphomicrobiaceae	Pedomicrobium	0,00%	0,07%	0,05%	0,02%	0,06%	0,04%	0,04%	0,00%	0,03%	0,04%
Bacteria	Proteobacteria	Alphaproteobacteria	Rhizobiales	Hyphomicrobiaceae	Rhodoplanes	0,01%	2,51%	2,83%	3,25%	2,72%	2,35%	2,03%	2,58%	2,24%	2,47%
Bacteria	Proteobacteria	Alphaproteobacteria	Rhizobiales	Methylobacteriaceae		1,29%	0,02%	0,03%	0,10%	0,03%	0,01%	0,00%	0,05%	0,02%	0,01%
Bacteria	Proteobacteria	Alphaproteobacteria	Rhizobiales	Methylobacteriaceae	Methylobacterium	3,91%	0,08%	0,09%	2,24%	0,07%	0,04%	0,03%	0,08%	0,05%	0,03%
Bacteria	Proteobacteria	Alphaproteobacteria	Rhizobiales	Methylocystaceae		0,00%	0,17%	0,19%	0,07%	0,14%	0,10%	0,08%	0,06%	0,10%	0,11%
Bacteria	Proteobacteria	Alphaproteobacteria	Rhizobiales	Methylocystaceae	Methylopila	0,00%	0,00%	0,00%	0,00%	0,00%	0,00%	0,00%	0,00%	0,00%	0,00%
Bacteria	Proteobacteria	Alphaproteobacteria	Rhizobiales	Phyllobacteriaceae		0,00%	0,19%	0,25%	0,91%	0,24%	0,18%	0,11%	0,10%	0,18%	0,19%
Bacteria	Proteobacteria	Alphaproteobacteria	Rhizobiales	Phyllobacteriaceae		0,00%	0,07%	0,09%	0,21%	0,08%	0,06%	0,03%	0,01%	0,06%	0,06%
Bacteria	Proteobacteria	Alphaproteobacteria	Rhizobiales	Phyllobacteriaceae	Aminobacter	0,00%	0,17%	0,22%	0,48%	0,24%	0,14%	0,13%	0,07%	0,12%	0,17%
Bacteria	Proteobacteria	Alphaproteobacteria	Rhizobiales	Phyllobacteriaceae	Mesorhizobium	0,00%	0,58%	0,98%	2,35%	0,83%	0,67%	0,39%	0,29%	0,58%	0,77%
Bacteria	Proteobacteria	Alphaproteobacteria	Rhizobiales	Phyllobacteriaceae	Phyllobacterium	0,00%	0,00%	0,00%	0,00%	0,00%	0,00%	0,00%	0,00%	0,00%	0,00%
Bacteria	Proteobacteria	Alphaproteobacteria	Rhizobiales	Rhizobiaceae		0,00%	0,05%	0,07%	0,07%	0,06%	0,04%	0,06%	0,05%	0,03%	0,04%
Bacteria	Proteobacteria	Alphaproteobacteria	Rhizobiales	Rhizobiaceae		0,00%	0,06%	0,02%	0,05%	0,06%	0,02%	0,03%	0,03%	0,02%	0,03%
Bacteria	Proteobacteria	Alphaproteobacteria	Rhizobiales	Rhizobiaceae	Agrobacterium	0,00%	0,02%	0,02%	0,04%	0,02%	0,01%	0,03%	0,02%	0,01%	0,01%
Bacteria	Proteobacteria	Alphaproteobacteria	Rhizobiales	Rhizobiaceae	Kaistia	0,00%	0,00%	0,00%	0,00%	0,00%	0,00%	0,00%	0,00%	0,00%	0,00%
Bacteria	Proteobacteria	Alphaproteobacteria	Rhizobiales	Rhizobiaceae	Rhizobium	0,00%	0,20%	0,43%	0,93%	0,30%	0,21%	0,27%	0,19%	0,19%	0,23%
Bacteria	Proteobacteria	Alphaproteobacteria	Rhizobiales	Rhodobiaceae		0,00%	0,00%	0,00%	0,00%	0,00%	0,00%	0,00%	0,00%	0,00%	0,00%
Bacteria	Proteobacteria	Alphaproteobacteria	Rhizobiales	Rhodobiaceae	Afifella	0,00%	0,20%	0,13%	0,16%	0,14%	0,09%	0,11%	0,02%	0,08%	0,10%
Bacteria	Proteobacteria	Alphaproteobacteria	Rhizobiales	Xanthobacteraceae		0,00%	0,00%	0,00%	0,00%	0,00%	0,00%	0,00%	0,00%	0,00%	0,00%
Bacteria	Proteobacteria	Alphaproteobacteria	Rhizobiales	Xanthobacteraceae	Labrys	0,00%	0,18%	0,22%	0,23%	0,18%	0,17%	0,08%	0,12%	0,17%	0,16%
Bacteria	Proteobacteria	Alphaproteobacteria	Rhizobiales	Xanthobacteraceae	Xanthobacter	0,00%	0,00%	0,00%	0,01%	0,00%	0,00%	0,00%	0,00%	0,00%	0,00%
Bacteria	Proteobacteria	Alphaproteobacteria	Rhodobacterales	Hyphomonadaceae		0,00%	0,04%	0,03%	0,01%	0,03%	0,04%	0,04%	0,00%	0,05%	0,04%
Bacteria	Proteobacteria	Alphaproteobacteria	Rhodobacterales	Rhodobacteraceae		0,00%	0,00%	0,00%	0,00%	0,00%	0,00%	0,00%	0,10%	0,00%	0,00%
Bacteria	Proteobacteria	Alphaproteobacteria	Rhodobacterales	Rhodobacteraceae		0,34%	0,01%	0,01%	0,01%	0,01%	0,01%	0,00%	0,01%	0,01%	0,01%
Bacteria	Proteobacteria	Alphaproteobacteria	Rhodobacterales	Rhodobacteraceae	Amaricoccus	0,00%	0,00%	0,00%	0,00%	0,00%	0,00%	0,00%	0,00%	0,00%	0,00%
Bacteria	Proteobacteria	Alphaproteobacteria	Rhodobacterales	Rhodobacteraceae	Paracoccus	0,01%	0,00%	0,00%	0,04%	0,00%	0,00%	0,00%	0,04%	0,00%	0,00%
Bacteria	Proteobacteria	Alphaproteobacteria	Rhodobacterales	Rhodobacteraceae	Rhodobacter	0,00%	0,00%	0,00%	0,00%	0,00%	0,00%	0,00%	0,00%	0,00%	0,00%
Bacteria	Proteobacteria	Alphaproteobacteria	Rhodobacterales	Rhodobacteraceae	Rubellimicrobium	0,00%	0,01%	0,01%	0,00%	0,00%	0,00%	0,00%	0,00%	0,00%	0,00%
Bacteria	Proteobacteria	Alphaproteobacteria	Rhodospirillales			0,00%	0,05%	0,05%	0,00%	0,07%	0,03%	0,02%	0,06%	0,03%	0,03%
Bacteria	Proteobacteria	Alphaproteobacteria	Rhodospirillales	Acetobacteraceae		0,18%	1,24%	1,05%	0,34%	1,07%	0,78%	0,50%	0,36%	0,75%	0,82%
Bacteria	Proteobacteria	Alphaproteobacteria	Rhodospirillales	Acetobacteraceae	Acidisoma	0,00%	0,03%	0,01%	0,01%	0,02%	0,02%	0,01%	0,00%	0,02%	0,02%
Bacteria	Proteobacteria	Alphaproteobacteria	Rhodospirillales	Acetobacteraceae	Acidocella	0,59%	0,00%	0,00%	0,00%	0,00%	0,00%	0,00%	0,00%	0,00%	0,00%
Bacteria	Proteobacteria	Alphaproteobacteria	Rhodospirillales	Acetobacteraceae	Roseomonas	0,00%	0,01%	0,01%	0,02%	0,01%	0,01%	0,00%	0,01%	0,00%	0,01%
Bacteria	Proteobacteria	Alphaproteobacteria	Rhodospirillales	Rhodospirillaceae		0,00%	1,94%	1,23%	2,25%	1,67%	1,51%	1,32%	0,70%	1,32%	1,75%
Bacteria	Proteobacteria	Alphaproteobacteria	Rhodospirillales	Rhodospirillaceae	Azospirillum	0,00%	0,00%	0,00%	0,00%	0,00%	0,00%	0,00%	0,00%	0,00%	0,00%
Bacteria	Proteobacteria	Alphaproteobacteria	Rhodospirillales	Rhodospirillaceae	Inquilinus	0,00%	0,00%	0,00%	0,00%	0,00%	0,00%	0,00%	0,00%	0,00%	0,00%
Bacteria	Proteobacteria	Alphaproteobacteria	Rhodospirillales	Rhodospirillaceae	Magnetospirillum	0,00%	0,00%	0,00%	0,00%	0,00%	0,00%	0,00%	0,00%	0,00%	0,00%
Bacteria	Proteobacteria	Alphaproteobacteria	Rhodospirillales	Rhodospirillaceae	Skermanella	0,00%	0,01%	0,01%	0,00%	0,00%	0,00%	0,00%	0,00%	0,01%	0,00%
Bacteria	Proteobacteria	Alphaproteobacteria	Rickettsiales			0,00%	0,12%	0,04%	1,19%	0,10%	0,05%	0,03%	0,30%	0,05%	0,06%

Bacteria	Proteobacteria	Alphaproteobacteria	Rickettsiales	Rickettsiaceae		0,00%	0,00%	0,00%	0,01%	0,00%	0,00%	0,00%	0,00%	0,00%	0,00%
Bacteria	Proteobacteria	Alphaproteobacteria	Rickettsiales	Rickettsiaceae	Rickettsia	0,00%	0,00%	0,00%	0,00%	0,00%	0,00%	0,00%	0,00%	0,00%	0,00%
Bacteria	Proteobacteria	Alphaproteobacteria	Rickettsiales	Rickettsiaceae	Wolbachia	0,00%	0,00%	0,00%	0,00%	0,00%	0,00%	0,00%	0,00%	0,00%	0,00%
Bacteria	Proteobacteria	Alphaproteobacteria	Rickettsiales	mitochondria		0,24%	0,03%	0,06%	0,00%	0,02%	0,04%	0,01%	0,01%	0,04%	0,03%
Bacteria	Proteobacteria	Alphaproteobacteria	Sphingomonadales	Other		0,00%	0,04%	0,02%	0,02%	0,02%	0,06%	0,04%	0,00%	0,05%	0,06%
Bacteria	Proteobacteria	Alphaproteobacteria	Sphingomonadales			0,00%	0,00%	0,00%	0,00%	0,00%	0,00%	0,00%	0,00%	0,00%	0,00%
Bacteria	Proteobacteria	Alphaproteobacteria	Sphingomonadales	Erythrobacteraceae		0,00%	0,00%	0,00%	0,00%	0,00%	0,00%	0,00%	0,00%	0,00%	0,00%
Bacteria	Proteobacteria	Alphaproteobacteria	Sphingomonadales	Erythrobacteraceae		0,00%	0,47%	0,28%	0,57%	0,33%	0,73%	0,82%	0,57%	0,70%	0,77%
Bacteria	Proteobacteria	Alphaproteobacteria	Sphingomonadales	Sphingomonadaceae		0,00%	0,02%	0,03%	0,09%	0,04%	0,02%	0,02%	0,00%	0,02%	0,02%
Bacteria	Proteobacteria	Alphaproteobacteria	Sphingomonadales	Sphingomonadaceae		0,05%	0,63%	0,54%	0,53%	0,57%	1,03%	0,94%	0,24%	1,02%	1,04%
Bacteria	Proteobacteria	Alphaproteobacteria	Sphingomonadales	Sphingomonadaceae	Blastomonas	0,00%	0,00%	0,00%	0,00%	0,00%	0,00%	0,00%	0,00%	0,00%	0,00%
Bacteria	Proteobacteria	Alphaproteobacteria	Sphingomonadales	Sphingomonadaceae	Kaistobacter	0,14%	6,79%	4,59%	23,68%	4,54%	19,58%	18,03%	48,91%	21,47%	17,34%
Bacteria	Proteobacteria	Alphaproteobacteria	Sphingomonadales	Sphingomonadaceae	Novosphingobium	0,00%	0,01%	0,01%	0,04%	0,01%	0,02%	0,02%	0,01%	0,03%	0,02%
Bacteria	Proteobacteria	Alphaproteobacteria	Sphingomonadales	Sphingomonadaceae	Sphingobium	0,00%	0,02%	0,02%	0,03%	0,01%	0,03%	0,03%	0,00%	0,03%	0,03%
Bacteria	Proteobacteria	Alphaproteobacteria	Sphingomonadales	Sphingomonadaceae	Sphingomonas	3,41%	1,12%	1,39%	1,62%	0,82%	1,85%	2,07%	0,53%	1,82%	1,88%
Bacteria	Proteobacteria	Alphaproteobacteria	Sphingomonadales	Sphingomonadaceae	Sphingopyxis	0,00%	0,02%	0,01%	0,02%	0,01%	0,02%	0,02%	0,00%	0,02%	0,03%
Bacteria	Proteobacteria	Betaproteobacteria	Other			0,00%	0,00%	0,00%	0,00%	0,00%	0,00%	0,00%	0,00%	0,00%	0,00%
Bacteria	Proteobacteria	Betaproteobacteria				0,00%	0,10%	0,03%	0,06%	0,06%	0,06%	0,06%	0,01%	0,06%	0,07%
Bacteria	Proteobacteria	Betaproteobacteria	A21b	EB1003		0,00%	0,37%	0,21%	0,08%	0,31%	0,33%	0,41%	0,07%	0,31%	0,34%
Bacteria	Proteobacteria	Betaproteobacteria	A21b	UD5		0,00%	0,08%	0,02%	0,01%	0,08%	0,05%	0,08%	0,00%	0,04%	0,05%
Bacteria	Proteobacteria	Betaproteobacteria	Burkholderiales			0,00%	0,01%	0,01%	0,00%	0,01%	0,02%	0,02%	0,01%	0,02%	0,01%
Bacteria	Proteobacteria	Betaproteobacteria	Burkholderiales	Alcaligenaceae		0,00%	0,01%	0,00%	0,02%	0,00%	0,01%	0,01%	0,03%	0,01%	0,01%
Bacteria	Proteobacteria	Betaproteobacteria	Burkholderiales	Alcaligenaceae	Achromobacter	0,00%	0,00%	0,00%	0,00%	0,00%	0,00%	0,00%	0,00%	0,00%	0,00%
Bacteria	Proteobacteria	Betaproteobacteria	Burkholderiales	Burkholderiaceae		0,00%	0,00%	0,00%	0,00%	0,00%	0,00%	0,00%	0,00%	0,00%	0,00%
Bacteria	Proteobacteria	Betaproteobacteria	Burkholderiales	Burkholderiaceae		0,00%	0,22%	0,35%	0,17%	0,17%	0,36%	0,28%	0,12%	0,39%	0,32%
Bacteria	Proteobacteria	Betaproteobacteria	Burkholderiales	Burkholderiaceae	Burkholderia	0,00%	1,46%	1,86%	1,36%	1,11%	1,67%	1,70%	0,60%	1,71%	1,62%
Bacteria	Proteobacteria	Betaproteobacteria	Burkholderiales	Comamonadaceae		0,00%	0,04%	0,04%	0,03%	0,04%	0,06%	0,07%	0,00%	0,06%	0,05%
Bacteria	Proteobacteria	Betaproteobacteria	Burkholderiales	Comamonadaceae		3,21%	0,16%	0,22%	0,12%	0,16%	0,23%	0,37%	0,44%	0,24%	0,22%
Bacteria	Proteobacteria	Betaproteobacteria	Burkholderiales	Comamonadaceae	Comamonas	0,00%	0,00%	0,00%	0,00%	0,00%	0,00%	0,00%	0,02%	0,00%	0,00%
Bacteria	Proteobacteria	Betaproteobacteria	Burkholderiales	Comamonadaceae	Delftia	0,04%	0,00%	0,00%	0,03%	0,00%	0,00%	0,00%	0,02%	0,00%	0,00%
Bacteria	Proteobacteria	Betaproteobacteria	Burkholderiales	Comamonadaceae	Methylibium	0,00%	0,10%	0,15%	0,04%	0,11%	0,21%	0,23%	0,04%	0,21%	0,21%
Bacteria	Proteobacteria	Betaproteobacteria	Burkholderiales	Comamonadaceae	Ramlibacter	0,00%	0,03%	0,02%	0,03%	0,02%	0,04%	0,04%	0,01%	0,04%	0,04%
Bacteria	Proteobacteria	Betaproteobacteria	Burkholderiales	Comamonadaceae	Rubrivivax	0,00%	0,00%	0,00%	0,00%	0,00%	0,00%	0,00%	0,00%	0,00%	0,00%
Bacteria	Proteobacteria	Betaproteobacteria	Burkholderiales	Comamonadaceae	Tepidimonas	0,28%	0,00%	0,00%	0,00%	0,00%	0,00%	0,00%	0,09%	0,00%	0,00%
Bacteria	Proteobacteria	Betaproteobacteria	Burkholderiales	Comamonadaceae	Variovorax	0,00%	0,00%	0,00%	0,00%	0,00%	0,01%	0,01%	0,01%	0,01%	0,01%
Bacteria	Proteobacteria	Betaproteobacteria	Burkholderiales	Oxalobacteraceae		0,00%	0,01%	0,01%	0,01%	0,01%	0,02%	0,15%	0,00%	0,02%	0,01%
Bacteria	Proteobacteria	Betaproteobacteria	Burkholderiales	Oxalobacteraceae		0,16%	0,13%	0,27%	0,09%	0,13%	0,34%	1,01%	0,08%	0,34%	0,33%
Bacteria	Proteobacteria	Betaproteobacteria	Burkholderiales	Oxalobacteraceae	Cupriavidus	0,00%	0,00%	0,00%	0,00%	0,00%	0,00%	0,00%	0,00%	0,00%	0,00%
Bacteria	Proteobacteria	Betaproteobacteria	Burkholderiales	Oxalobacteraceae	Janthinobacterium	0,00%	0,01%	0,04%	0,02%	0,01%	0,05%	0,22%	0,04%	0,04%	0,05%
Bacteria	Proteobacteria	Betaproteobacteria	Burkholderiales	Oxalobacteraceae	Ralstonia	0,00%	0,00%	0,00%	0,00%	0,01%	0,00%	0,00%	0,00%	0,00%	0,00%
Bacteria	Proteobacteria	Betaproteobacteria	Ellin6067			0,00%	0,25%	0,08%	0,30%	0,18%	0,22%	0,25%	0,05%	0,20%	0,23%
Bacteria	Proteobacteria	Betaproteobacteria	Hydrogenophilales	Hydrogenophilaceae	Thiobacillus	0,00%	0,00%	0,00%	0,00%	0,00%	0,00%	0,00%	0,00%	0,00%	0,00%
Bacteria	Proteobacteria	Betaproteobacteria	MND1			0,00%	0,12%	0,06%	0,05%	0,10%	0,10%	0,09%	0,00%	0,11%	0,09%
Bacteria	Proteobacteria	Betaproteobacteria	Methylophilales	Methylophilaceae		0,00%	0,00%	0,00%	0,01%	0,00%	0,00%	0,00%	0,00%	0,00%	0,00%
Bacteria	Proteobacteria	Betaproteobacteria	Neisseriales	Neisseriaceae		0,36%	0,00%	0,00%	0,00%	0,00%	0,00%	0,00%	0,00%	0,00%	0,00%
Bacteria	Proteobacteria	Betaproteobacteria	Nitrosomonadales	Nitrosomonadaceae		0,00%	0,00%	0,00%	0,00%	0,00%	0,00%	0,00%	0,00%	0,00%	0,00%
Bacteria	Proteobacteria	Betaproteobacteria	Rhodocyclales	Rhodocyclaceae		0,00%	0,00%	0,00%	0,00%	0,00%	0,00%	0,00%	0,00%	0,00%	0,00%
Bacteria	Proteobacteria	Betaproteobacteria	Rhodocyclales	Rhodocyclaceae		0,00%	0,00%	0,00%	0,00%	0,00%	0,00%	0,00%	0,00%	0,00%	0,00%
Bacteria	Proteobacteria	Betaproteobacteria	Rhodocyclales	Rhodocyclaceae	Hydrogenophilus	0,04%	0,00%	0,00%	0,00%	0,00%	0,00%	0,00%	0,00%	0,00%	0,00%
Bacteria	Proteobacteria	Betaproteobacteria	Rhodocyclales	Rhodocyclaceae	Zoogloea	0,00%	0,00%	0,00%	0,00%	0,00%	0,00%	0,00%	0,01%	0,00%	0,00%
Bacteria	Proteobacteria	Betaproteobacteria	SC-I-84			0,00%	0,80%	0,32%	0,10%	0,65%	0,59%	0,75%	0,09%	0,56%	0,62%
Bacteria	Proteobacteria	Deltaproteobacteria				0,00%	0,00%	0,00%	0,00%	0,00%	0,00%	0,00%	0,00%	0,00%	0,00%
Bacteria	Proteobacteria	Deltaproteobacteria	Bdellovibrionales	Bacteriovoracaceae		0,00%	0,01%	0,01%	0,00%	0,00%	0,01%	0,01%	0,02%	0,01%	0,01%

Bacteria	Proteobacteria	Deltaproteobacteria	Bdellovibrionales	Bdellovibrionaceae	Bdellovibrio	0,00%	0,11%	0,06%	0,02%	0,06%	0,08%	0,11%	0,04%	0,08%	0,09%
Bacteria	Proteobacteria	Deltaproteobacteria	MIZ46			0,00%	0,03%	0,01%	0,00%	0,02%	0,02%	0,02%	0,01%	0,02%	0,02%
Bacteria	Proteobacteria	Deltaproteobacteria	Myxococcales	Other		0,00%	0,01%	0,02%	0,00%	0,01%	0,01%	0,01%	0,00%	0,01%	0,01%
Bacteria	Proteobacteria	Deltaproteobacteria	Myxococcales			0,00%	0,87%	1,07%	0,29%	0,97%	0,72%	1,78%	0,22%	0,66%	0,80%
Bacteria	Proteobacteria	Deltaproteobacteria	Myxococcales	0319-6G20		0,00%	0,03%	0,01%	0,02%	0,02%	0,02%	0,03%	0,01%	0,02%	0,02%
Bacteria	Proteobacteria	Deltaproteobacteria	Myxococcales	Cystobacterineae		0,00%	0,00%	0,00%	0,00%	0,00%	0,00%	0,00%	0,00%	0,00%	0,00%
Bacteria	Proteobacteria	Deltaproteobacteria	Myxococcales	Haliangiaceae		0,00%	0,43%	0,27%	0,03%	0,36%	0,37%	0,28%	0,03%	0,36%	0,39%
Bacteria	Proteobacteria	Deltaproteobacteria	Myxococcales	Myxococcaceae		0,00%	0,01%	0,00%	0,00%	0,00%	0,00%	0,01%	0,00%	0,00%	0,00%
Bacteria	Proteobacteria	Deltaproteobacteria	Myxococcales	Myxococcaceae	Myxococcus	0,00%	0,00%	0,00%	0,00%	0,00%	0,00%	0,00%	0,00%	0,00%	0,00%
Bacteria	Proteobacteria	Deltaproteobacteria	Myxococcales	Nannocystaceae	Nannocystis	0,00%	0,00%	0,00%	0,00%	0,00%	0,00%	0,00%	0,00%	0,00%	0,00%
Bacteria	Proteobacteria	Deltaproteobacteria	Myxococcales	OM27		0,00%	0,00%	0,00%	0,00%	0,00%	0,00%	0,00%	0,00%	0,00%	0,00%
Bacteria	Proteobacteria	Deltaproteobacteria	Myxococcales	Polyangiaceae		0,00%	0,00%	0,00%	0,00%	0,00%	0,00%	0,00%	0,00%	0,00%	0,00%
Bacteria	Proteobacteria	Deltaproteobacteria	Spirobacillales			0,00%	0,01%	0,01%	0,00%	0,01%	0,01%	0,00%	0,00%	0,01%	0,01%
Bacteria	Proteobacteria	Deltaproteobacteria	Syntrophobacterales	Syntrophobacteraceae		0,00%	0,13%	0,08%	0,02%	0,10%	0,10%	0,10%	0,02%	0,10%	0,10%
Bacteria	Proteobacteria	Gammaproteobacteria				0,00%	0,00%	0,00%	0,00%	0,01%	0,00%	0,00%	0,00%	0,00%	0,00%
Bacteria	Proteobacteria	Gammaproteobacteria	Aeromonadales	Aeromonadaceae		0,00%	0,00%	0,00%	0,01%	0,00%	0,00%	0,00%	0,00%	0,00%	0,00%
Bacteria	Proteobacteria	Gammaproteobacteria	Alteromonadales	211ds20		0,00%	0,00%	0,00%	0,00%	0,00%	0,00%	0,00%	0,00%	0,00%	0,00%
Bacteria	Proteobacteria	Gammaproteobacteria	Alteromonadales	Alteromonadaceae	Cellvibrio	0,00%	0,00%	0,03%	0,00%	0,01%	0,01%	0,04%	0,01%	0,00%	0,01%
Bacteria	Proteobacteria	Gammaproteobacteria	Alteromonadales	Alteromonadaceae	HB2-32-21	0,00%	0,00%	0,00%	0,01%	0,00%	0,00%	0,00%	0,02%	0,00%	0,00%
Bacteria	Proteobacteria	Gammaproteobacteria	Alteromonadales	Alteromonadaceae	Marinobacter	4,35%	0,00%	0,00%	0,00%	0,00%	0,00%	0,00%	0,02%	0,00%	0,00%
Bacteria	Proteobacteria	Gammaproteobacteria	Alteromonadales	[Chromatiaceae]	Rheinheimera	0,00%	0,00%	0,00%	0,00%	0,00%	0,00%	0,00%	0,00%	0,00%	0,00%
Bacteria	Proteobacteria	Gammaproteobacteria	Chromatiales	Other		0,00%	0,16%	0,38%	0,02%	0,36%	0,42%	0,28%	0,07%	0,40%	0,45%
Bacteria	Proteobacteria	Gammaproteobacteria	Chromatiales			0,00%	0,00%	0,00%	0,00%	0,00%	0,00%	0,00%	0,00%	0,00%	0,00%
Bacteria	Proteobacteria	Gammaproteobacteria	Chromatiales	Ectothiorhodospiraceae		0,00%	0,07%	0,04%	0,02%	0,09%	0,16%	0,06%	0,05%	0,14%	0,19%
Bacteria	Proteobacteria	Gammaproteobacteria	Enterobacteriales	Enterobacteriaceae		0,00%	0,00%	0,00%	0,01%	0,00%	0,00%	0,00%	0,00%	0,00%	0,00%
Bacteria	Proteobacteria	Gammaproteobacteria	Enterobacteriales	Enterobacteriaceae		0,00%	0,00%	0,00%	0,03%	0,00%	0,00%	0,00%	0,04%	0,00%	0,00%
Bacteria	Proteobacteria	Gammaproteobacteria	Enterobacteriales	Enterobacteriaceae	Enterobacter	0,00%	0,00%	0,00%	0,00%	0,00%	0,00%	0,00%	0,00%	0,00%	0,00%
Bacteria	Proteobacteria	Gammaproteobacteria	Enterobacteriales	Enterobacteriaceae	Erwinia	0,00%	0,00%	0,00%	0,03%	0,00%	0,00%	0,00%	0,01%	0,00%	0,00%
Bacteria	Proteobacteria	Gammaproteobacteria	Enterobacteriales	Enterobacteriaceae	Serratia	0,00%	0,00%	0,00%	0,00%	0,00%	0,00%	0,00%	0,00%	0,00%	0,00%
Bacteria	Proteobacteria	Gammaproteobacteria	Legionellales			0,00%	0,00%	0,00%	0,00%	0,01%	0,00%	0,00%	0,00%	0,00%	0,00%
Bacteria	Proteobacteria	Gammaproteobacteria	Legionellales	Coxiellaceae		0,00%	0,03%	0,02%	0,47%	0,03%	0,04%	0,03%	0,75%	0,03%	0,05%
Bacteria	Proteobacteria	Gammaproteobacteria	Legionellales	Coxiellaceae	Aquicella	0,00%	0,02%	0,02%	0,64%	0,02%	0,03%	0,02%	0,77%	0,02%	0,03%
Bacteria	Proteobacteria	Gammaproteobacteria	Legionellales	Coxiellaceae	Rickettsiella	0,00%	0,00%	0,00%	0,00%	0,00%	0,00%	0,00%	0,02%	0,00%	0,00%
Bacteria	Proteobacteria	Gammaproteobacteria	Legionellales	Legionellaceae		0,00%	0,00%	0,00%	0,01%	0,00%	0,00%	0,00%	0,00%	0,00%	0,00%
Bacteria	Proteobacteria	Gammaproteobacteria	Legionellales	Legionellaceae		0,04%	0,01%	0,00%	0,04%	0,00%	0,00%	0,00%	0,01%	0,00%	0,00%
Bacteria	Proteobacteria	Gammaproteobacteria	Legionellales	Legionellaceae	Legionella	1,70%	0,04%	0,03%	0,48%	0,04%	0,01%	0,01%	0,20%	0,01%	0,01%
Bacteria	Proteobacteria	Gammaproteobacteria	Legionellales	Legionellaceae	Tatlockia	0,00%	0,00%	0,00%	0,05%	0,00%	0,00%	0,00%	0,00%	0,00%	0,00%
Bacteria	Proteobacteria	Gammaproteobacteria	Oceanospirillales	Alcanivoracaceae	Alcanivorax	0,00%	0,00%	0,00%	0,00%	0,00%	0,00%	0,00%	0,00%	0,00%	0,00%
Bacteria	Proteobacteria	Gammaproteobacteria	Oceanospirillales	Halomonadaceae	Chromohalobacter	0,53%	0,00%	0,00%	0,00%	0,00%	0,00%	0,00%	0,00%	0,00%	0,00%
Bacteria	Proteobacteria	Gammaproteobacteria	Oceanospirillales	Halomonadaceae	Halomonas	0,97%	0,00%	0,00%	0,00%	0,00%	0,00%	0,00%	0,01%	0,00%	0,00%
Bacteria	Proteobacteria	Gammaproteobacteria	PYR10d3			0,00%	0,03%	0,01%	0,00%	0,03%	0,03%	0,02%	0,00%	0,03%	0,03%
Bacteria	Proteobacteria	Gammaproteobacteria	Pasteurellales	Pasteurellaceae	Actinobacillus	0,00%	0,00%	0,00%	0,00%	0,00%	0,00%	0,00%	0,00%	0,00%	0,00%
Bacteria	Proteobacteria	Gammaproteobacteria	Pasteurellales	Pasteurellaceae	Haemophilus	0,00%	0,00%	0,00%	0,00%	0,00%	0,00%	0,00%	0,02%	0,00%	0,00%
Bacteria	Proteobacteria	Gammaproteobacteria	Pseudomonadales	Moraxellaceae		0,00%	0,00%	0,00%	0,04%	0,00%	0,00%	0,00%	0,00%	0,00%	0,00%
Bacteria	Proteobacteria	Gammaproteobacteria	Pseudomonadales	Moraxellaceae	Acinetobacter	1,73%	0,00%	0,00%	0,09%	0,00%	0,00%	0,02%	0,64%	0,00%	0,01%
Bacteria	Proteobacteria	Gammaproteobacteria	Pseudomonadales	Moraxellaceae	Alkanindiges	3,18%	0,00%	0,00%	0,00%	0,00%	0,00%	0,00%	0,00%	0,00%	0,00%
Bacteria	Proteobacteria	Gammaproteobacteria	Pseudomonadales	Moraxellaceae	Enhydrobacter	0,00%	0,00%	0,00%	0,56%	0,00%	0,00%	0,00%	0,02%	0,00%	0,00%
Bacteria	Proteobacteria	Gammaproteobacteria	Pseudomonadales	Moraxellaceae	Perlucidibaca	0,00%	0,00%	0,00%	0,00%	0,00%	0,00%	0,00%	0,00%	0,00%	0,00%
Bacteria	Proteobacteria	Gammaproteobacteria	Pseudomonadales	Pseudomonadaceae		0,00%	0,00%	0,00%	0,00%	0,00%	0,00%	0,00%	0,00%	0,00%	0,00%
Bacteria	Proteobacteria	Gammaproteobacteria	Pseudomonadales	Pseudomonadaceae		0,00%	0,00%	0,00%	0,00%	0,00%	0,00%	0,00%	0,00%	0,00%	0,00%
Bacteria	Proteobacteria	Gammaproteobacteria	Pseudomonadales	Pseudomonadaceae	Pseudomonas	1,08%	0,05%	0,15%	0,63%	0,04%	0,16%	0,21%	0,04%	0,16%	0,15%
Bacteria	Proteobacteria	Gammaproteobacteria	Salinisphaerales	Salinisphaeraceae	Salinisphaera	0,15%	0,00%	0,00%	0,00%	0,00%	0,00%	0,00%	0,00%	0,00%	0,00%
Bacteria	Proteobacteria	Gammaproteobacteria	Thiotrichales	Piscirickettsiaceae		0,00%	0,03%	0,03%	0,01%	0,02%	0,02%	0,02%	0,01%	0,03%	0,02%

Bacteria	Proteobacteria	Gammaproteobacteria	Xanthomonadales	Sinobacteraceae		0,00%	0,44%	0,98%	0,23%	0,50%	0,57%	0,80%	0,20%	0,58%	0,56%
Bacteria	Proteobacteria	Gammaproteobacteria	Xanthomonadales	Sinobacteraceae	Steroidobacter	0,00%	0,02%	0,06%	0,01%	0,04%	0,03%	0,04%	0,02%	0,04%	0,03%
Bacteria	Proteobacteria	Gammaproteobacteria	Xanthomonadales	Xanthomonadaceae		0,00%	0,15%	0,21%	0,27%	0,12%	0,38%	1,03%	0,08%	0,38%	0,37%
Bacteria	Proteobacteria	Gammaproteobacteria	Xanthomonadales	Xanthomonadaceae		0,00%	1,84%	1,82%	1,71%	1,50%	3,16%	5,37%	0,90%	3,07%	3,25%
Bacteria	Proteobacteria	Gammaproteobacteria	Xanthomonadales	Xanthomonadaceae	Dokdonella	0,00%	0,35%	0,73%	0,24%	0,58%	0,80%	0,64%	0,15%	0,88%	0,71%
Bacteria	Proteobacteria	Gammaproteobacteria	Xanthomonadales	Xanthomonadaceae	Luteibacter	0,00%	0,00%	0,01%	0,01%	0,00%	0,01%	0,02%	0,00%	0,01%	0,01%
Bacteria	Proteobacteria	Gammaproteobacteria	Xanthomonadales	Xanthomonadaceae	Luteimonas	0,00%	0,02%	0,02%	0,05%	0,02%	0,04%	0,11%	0,01%	0,04%	0,04%
Bacteria	Proteobacteria	Gammaproteobacteria	Xanthomonadales	Xanthomonadaceae	Lysobacter	0,00%	0,01%	0,01%	0,02%	0,01%	0,03%	0,03%	0,01%	0,03%	0,02%
Bacteria	Proteobacteria	Gammaproteobacteria	Xanthomonadales	Xanthomonadaceae	Rhodanobacter	0,00%	0,24%	0,63%	0,62%	0,25%	0,62%	1,93%	0,15%	0,67%	0,55%
Bacteria	Proteobacteria	Gammaproteobacteria	Xanthomonadales	Xanthomonadaceae	Stenotrophomonas	0,00%	0,00%	0,00%	0,02%	0,00%	0,00%	0,01%	0,01%	0,01%	0,00%
Bacteria	Proteobacteria	Gammaproteobacteria	Xanthomonadales	Xanthomonadaceae	Xanthomonas	0,00%	0,00%	0,00%	0,00%	0,00%	0,00%	0,00%	0,00%	0,00%	0,00%
Bacteria	Proteobacteria	TA18	CV90			0,00%	0,00%	0,00%	0,00%	0,00%	0,00%	0,00%	0,00%	0,00%	0,00%
Bacteria	Proteobacteria	TA18	PHOS-HD29			0,00%	0,00%	0,00%	0,01%	0,00%	0,00%	0,00%	0,00%	0,00%	0,00%
Bacteria	Spirochaetes	Spirochaetes	Spirochaetales	Spirochaetaceae	Spirochaeta	0,00%	0,00%	0,00%	0,00%	0,00%	0,00%	0,00%	0,00%	0,00%	0,00%
Bacteria	TM6	SJA-4				0,00%	0,14%	0,10%	0,29%	0,10%	0,09%	0,09%	0,16%	0,09%	0,08%
Bacteria	TM6	SJA-4	S1198			0,00%	0,00%	0,00%	0,01%	0,00%	0,00%	0,00%	0,00%	0,00%	0,00%
Bacteria	TM6	SJA-4	YJF2-48			0,00%	0,00%	0,00%	0,00%	0,00%	0,00%	0,00%	0,00%	0,00%	0,00%
Bacteria	TM7					0,00%	0,00%	0,00%	0,02%	0,00%	0,00%	0,00%	0,03%	0,00%	0,00%
Bacteria	TM7	MJK10				0,00%	0,00%	0,00%	0,00%	0,00%	0,00%	0,00%	0,00%	0,00%	0,00%
Bacteria	TM7	SC3				0,00%	0,03%	0,02%	0,08%	0,03%	0,05%	0,07%	0,13%	0,05%	0,05%
Bacteria	TM7	TM7-1				0,00%	0,35%	0,20%	1,66%	0,28%	0,46%	0,46%	0,97%	0,50%	0,43%
Bacteria	TM7	TM7-3				0,00%	0,07%	0,06%	0,57%	0,07%	0,12%	0,12%	0,61%	0,12%	0,11%
Bacteria	TM7	TM7-3	EW055			0,00%	0,08%	0,04%	0,18%	0,07%	0,10%	0,15%	0,40%	0,11%	0,09%
Bacteria	TM7	TM7-3	I025			0,00%	0,02%	0,01%	0,03%	0,01%	0,03%	0,02%	0,01%	0,03%	0,02%
Bacteria	Tenericutes	Mollicutes	Anaeroplasmatales	Anaeroplasmataceae		0,00%	0,00%	0,00%	0,00%	0,00%	0,00%	0,01%	0,00%	0,00%	0,00%
Bacteria	Tenericutes	Mollicutes	Anaeroplasmatales	Anaeroplasmataceae	Asteroleplasma	0,00%	0,01%	0,03%	0,00%	0,01%	0,01%	0,01%	0,00%	0,01%	0,01%
Bacteria	Verrucomicrobia	Opitutae				0,00%	0,00%	0,00%	0,00%	0,00%	0,00%	0,00%	0,00%	0,00%	0,00%
Bacteria	Verrucomicrobia	Opitutae	Opitutales	Opitutaceae		0,00%	0,03%	0,03%	0,04%	0,02%	0,03%	0,06%	0,01%	0,04%	0,03%
Bacteria	Verrucomicrobia	Opitutae	Opitutales	Opitutaceae	Opitutus	0,00%	0,26%	0,16%	0,08%	0,18%	0,23%	0,29%	0,11%	0,23%	0,22%
Bacteria	Verrucomicrobia	Verrucomicrobiae	Verrucomicrobiales	Verrucomicrobiaceae		0,00%	0,01%	0,01%	0,01%	0,00%	0,01%	0,00%	0,00%	0,00%	0,01%
Bacteria	Verrucomicrobia	Verrucomicrobiae	Verrucomicrobiales	Verrucomicrobiaceae	Luteolibacter	0,00%	0,01%	0,01%	0,00%	0,01%	0,01%	0,02%	0,01%	0,01%	0,01%
Bacteria	Verrucomicrobia	Verrucomicrobiae	Verrucomicrobiales	Verrucomicrobiaceae	Prosthecobacter	0,00%	0,00%	0,00%	0,00%	0,00%	0,00%	0,00%	0,01%	0,00%	0,00%
Bacteria	Verrucomicrobia	[Methylacidiphilae]	Methylacidiphilales			0,00%	0,01%	0,00%	0,00%	0,01%	0,01%	0,01%	0,00%	0,01%	0,01%
Bacteria	Verrucomicrobia	[Pedosphaerae]	[Pedosphaerales]			0,00%	0,18%	0,11%	0,03%	0,11%	0,13%	0,15%	0,05%	0,13%	0,15%
Bacteria	Verrucomicrobia	[Pedosphaerae]	[Pedosphaerales]	Ellin515		0,00%	0,12%	0,03%	0,02%	0,07%	0,06%	0,05%	0,02%	0,06%	0,06%
Bacteria	Verrucomicrobia	[Pedosphaerae]	[Pedosphaerales]	Ellin517		0,00%	0,03%	0,01%	0,01%	0,02%	0,01%	0,01%	0,00%	0,02%	0,01%
Bacteria	Verrucomicrobia	[Pedosphaerae]	[Pedosphaerales]	auto67	4W	0,00%	0,19%	0,13%	0,02%	0,10%	0,16%	0,12%	0,00%	0,16%	0,16%
Bacteria	Verrucomicrobia	[Spartobacteria]	[Chthoniobacterales]	01D2Z36		0,00%	0,00%	0,00%	0,00%	0,00%	0,00%	0,00%	0,00%	0,00%	0,00%
Bacteria	Verrucomicrobia	[Spartobacteria]	[Chthoniobacterales]	[Chthoniobacteraceae]		0,00%	0,01%	0,00%	0,00%	0,01%	0,00%	0,01%	0,00%	0,00%	0,01%
Bacteria	Verrucomicrobia	[Spartobacteria]	[Chthoniobacterales]	[Chthoniobacteraceae]	Candidatus Xiphinematobacter	0,00%	0,05%	0,03%	0,16%	0,03%	0,03%	0,02%	0,03%	0,03%	0,03%
Bacteria	Verrucomicrobia	[Spartobacteria]	[Chthoniobacterales]	[Chthoniobacteraceae]	Chthoniobacter	0,00%	0,01%	0,00%	0,01%	0,01%	0,01%	0,01%	0,00%	0,01%	0,01%
Bacteria	Verrucomicrobia	[Spartobacteria]	[Chthoniobacterales]	[Chthoniobacteraceae]	DA101	0,00%	0,55%	0,49%	0,24%	0,37%	0,45%	0,61%	0,10%	0,46%	0,44%
Bacteria	Verrucomicrobia	[Spartobacteria]	[Chthoniobacterales]	[Chthoniobacteraceae]	Ellin506	0,00%	0,00%	0,00%	0,00%	0,00%	0,00%	0,00%	0,00%	0,00%	0,00%
Bacteria	Verrucomicrobia	[Spartobacteria]	[Chthoniobacterales]	[Chthoniobacteraceae]	OR-59	0,00%	0,00%	0,00%	0,00%	0,00%	0,00%	0,00%	0,00%	0,00%	0,00%
Bacteria	WPS-2					0,00%	0,15%	0,13%	1,39%	0,22%	0,43%	0,43%	0,55%	0,34%	0,52%
Bacteria	WS2	SHA-109				0,00%	0,01%	0,00%	0,03%	0,01%	0,01%	0,01%	0,00%	0,02%	0,01%
Bacteria	[Thermi]	Deinococci	Deinococcales	Deinococcaceae	Deinococcus	0,00%	0,01%	0,03%	0,00%	0,01%	0,02%	0,01%	0,02%	0,02%	0,01%
Bacteria	[Thermi]	Deinococci	Thermales	Thermaceae	Thermus	6,97%	0,00%	0,00%	0,00%	0,00%	0,00%	0,00%	0,03%	0,00%	0,00%
