# Supplementary material for: The choice of the DNA extraction method may influence the outcome of the soil microbial community structure analysis
Source: Microbiologyopen. 2017 Feb 20;6(4):e00453. doi: 10.1002/mbo3.453 (PMC5552907; doi:10.1002/mbo3.453)
Supplement: Supplementary file 3 [file MBO3-6-na-s003.pdf]

Supplementary Table 3. Statistical differences among each phyla between extraction protocols characterized by the Kruskla-Wallis test and p-values.

Phylum	Kruskla-Wallis	p-value
Actinobacteria	24.78042328	0.000829884
Planctomycetes	24.74714751	0.000841211
TM7	24.49424936	0.000932386
Verrucomicrobia	24.06286997	0.001110718
WPS-2	23.52532355	0.001380106
Bacteroidetes	22.68518519	0.001933587
Proteobacteria	22.12698413	0.002415454
Tenericutes	21.7247765	0.002833225
Firmicutes	21.53439153	0.003054719
Nitrospirae	21.22794194	0.003447034
Chloroflexi	20.88468193	0.003944628
Chlorobi	20.8753216	0.003959129
Gemmatimonadetes	20.65974555	0.004307776
Armatimonadetes	19.84905564	0.005905115
Acidobacteria	19.61640212	0.00646066
Crenarchaeota	18.10630631	0.011499466
TM6	18.01506332	0.011902224
OD1	17.84642127	0.012682471
Chlamydiae	17.10534887	0.016729357
OP11	16.84874389	0.018397981
Elusimicrobia	16.48509485	0.021035743
FBP	15.91037687	0.025947903
MVP-21	14.84088778	0.038093946
WS2	14.25932364	0.046756641
Fibrobacteres	13.89067254	0.053160367
Cyanobacteria	13.01984733	0.071626289
AD3	9.982637583	0.189559517
Fusobacteria	8	0.332593903
[Thermi]	7.320656111	0.396273917
Euryarchaeota	7.282051282	0.400115405
BRC1	7.282051282	0.400115405
Spirochaetes	7.28	0.400320178
